# Specific Evolution of F_1_-Like ATPases in Mycoplasmas

**DOI:** 10.1371/journal.pone.0038793

**Published:** 2012-06-07

**Authors:** Laure Béven, Claire Charenton, Alain Dautant, Guillaume Bouyssou, Fabien Labroussaa, Anna Sköllermo, Anja Persson, Alain Blanchard, Pascal Sirand-Pugnet

**Affiliations:** 1 University Bordeaux, UMR 1332 de Biologie du Fruit et Pathologie, Villenave d'Ornon, France; 2 INRA, UMR 1332 de Biologie du Fruit et Pathologie, Villenave d'Ornon, France; 3 University Bordeaux, IBGC, UMR 5095, Bordeaux, France; 4 CNRS, IBGC, UMR 5095, Bordeaux, France; 5 Department of Proteomics, School of Biotechnology, KTH-Royal Institute of Technology, AlbaNova University Center, Stockholm, Sweden; Institut de Pharmacologie et de Biologie Structurale, France

## Abstract

F_1_F_0_ ATPases have been identified in most bacteria, including mycoplasmas which have very small genomes associated with a host-dependent lifestyle. In addition to the typical operon of eight genes encoding genuine F_1_F_0_ ATPase (Type 1), we identified related clusters of seven genes in many mycoplasma species. Four of the encoded proteins have predicted structures similar to the α, β, γ and ε subunits of F_1_ ATPases and could form an F_1_-like ATPase. The other three proteins display no similarity to any other known proteins. Two of these proteins are probably located in the membrane, as they have three and twelve predicted transmembrane helices. Phylogenomic studies identified two types of F_1_-like ATPase clusters, Type 2 and Type 3, characterized by a rapid evolution of sequences with the conservation of structural features. Clusters encoding Type 2 and Type 3 ATPases were assumed to originate from the Hominis group of mycoplasmas. We suggest that Type 3 ATPase clusters may spread to other phylogenetic groups by horizontal gene transfer between mycoplasmas in the same host, based on phylogeny and genomic context. Functional analyses in the ruminant pathogen *Mycoplasma mycoides* subsp. *mycoides* showed that the Type 3 cluster genes were organized into an operon. Proteomic analyses demonstrated that the seven encoded proteins were produced during growth in axenic media. Mutagenesis and complementation studies demonstrated an association of the Type 3 cluster with a major ATPase activity of membrane fractions. Thus, despite their tendency toward genome reduction, mycoplasmas have evolved and exchanged specific F_1_-like ATPases with no known equivalent in other bacteria. We propose a model, in which the F_1_-like structure is associated with a hypothetical X_0_ sector located in the membrane of mycoplasma cells.

## Introduction

Mycoplasmas are small bacteria that infect humans and animals and evolved from low-GC content firmicutes in a process involving a drastic reduction of genome size, resulting in present-day species with typical 1 Mb-genomes [1]. Mycoplasmas have lost genes from most functional categories and display the complete disappearance of several metabolic pathways and the elimination of many redundant genes. Like other members of the class *Mollicutes*, the bacteria of the *Mycoplasma* genus lack genes involved in the synthesis of cell-wall components, amino-acids, lipids, co-factors and nucleic acid precursors.

The cellular apparatus involved in the basic maintenance and expression of genetic information is essentially similar in most mycoplasmas [2], but the enzymes involved in energy metabolism may differ considerably between, even in those with very reduced genomes [3]. The repertoires of genes encoding membrane proteins such as lipoproteins and transporters are also highly diverse in mycoplasmas, probably reflecting the ability of the different species to infect animal species as diverse as mammals, birds, fishes and arthropods. Thus, despite the massive genome reduction that has marked their evolution and their general absence in natural environments, mycoplasmas have conquered a wide range of complex animals and seem to be able to adapt rapidly to new hosts. Phylogenomic studies based on 16S rDNA and other genes have shown that mycoplasmas are frequently associated with particularly long branches [4]. Moreover, going against the widespread view that mycoplasmas evolve purely by gene loss, recent studies have shown that horizontal gene transfers (HGT) between species in the same host may have increased the genetic potential of mycoplasmas, potentially facilitating adaptation to the host. Three examples of HGT have been reported to date, in mycoplasmas pathogenic to humans [3], birds [5] and ruminants [6]. In the species infecting birds and ruminants, several genes thought to have been subject to HGT were parts of typical mobile elements including integrative conjugative elements (ICEs), insertion sequences (ISs) and restriction-modification systems (RMSs) but many others encoded transporters, lipoproteins and hypothetical proteins potentially involved in host-specificity and pathogenicity. The genes thought to have been subject to HGT in human urogenital species encoded ISs, RMSs, hypothetical proteins and two proteins related to F_1_F_0_ ATPase subunits α (*atpA*) and β (*atpD*). Strikingly, genes annotated *atpA* and *atpD* were also found in the lists of genes thought to have been exchanged between bird mycoplasma species and between ruminant mycoplasma species.

**Figure 1:**
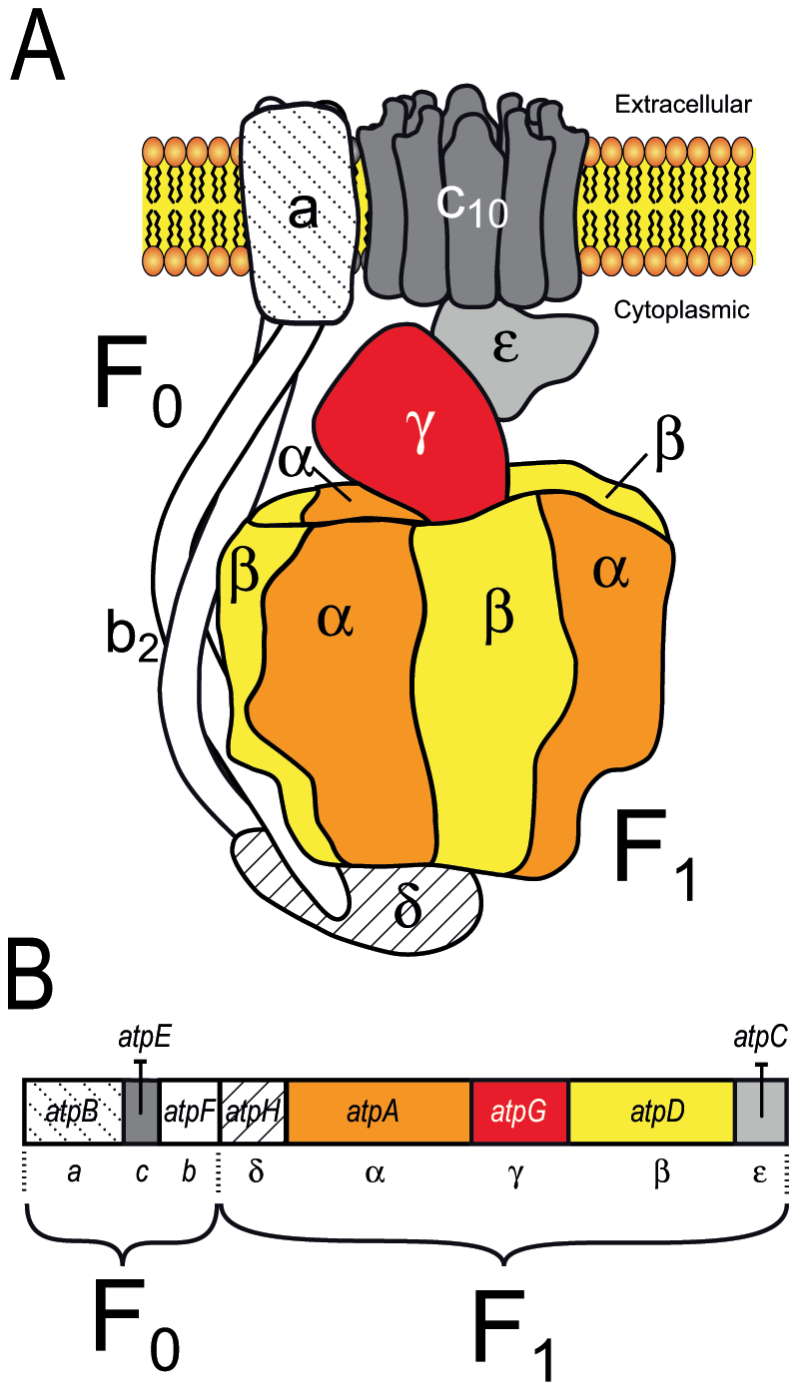
ATPase F_1_F_0_ in mycoplasmas. **A**. Bacterial ATPase F_1_F_0_. **B**. Organization of the operon encoding the ATPase F_1_F_0_ in mycoplasmas. In *E. coli* and mycoplasma species, the F_1_F_0_ ATPase operon and likely the 3D structure are similar.

All the mycoplasma genomes examined to date contain a typical complete operon encoding the eight subunits of the F_1_F_0_ ATPase (**Figure 1**). The F_1_F_0_ ATPase is thought to function primarily in ATP hydrolysis and maintenance of the electrochemical gradient in mycoplasmas, rather than in the generation of ATP [7]. Nevertheless, the genes encoding the subunits of this complex were considered to be essential in several species in which global transposon mutagenesis was carried out [8], [9], [10]. Surprisingly, in addition to the F_1_F_0_ ATPase operon, extra copies of *atpA* and *atpD*, organized in pairs, have been identified and annotated in many mycoplasma genomes although no specific study has ever investigated this remarkable trait. Indeed, although most bacteria have F_1_F_0_ ATPases, the presence of extra genes located outside of the traditional operon has been reported in very few cases including those encoding atypical forms of F_1_F_0_ ATPase (N-ATPases) in some marine and halotolerant bacteria, in the pathogens *Burkolderia* spp. and in the archaea *Methanosarcina acetivorans* and *Methanosarcina barkeri* [11]. Thus, in the general context of gene loss and redundancy elimination during the evolution of mycoplasmas, the presence of additional copies was entirely unexpected. Moreover, the prediction that these extra copies of *atpA* and *atpD* may have been exchanged during three unrelated HGT events between mycoplasmas was puzzling.

We investigated the evolution of these genes and their relationship to the F_1_F_0_ ATPase, by carrying out a global phylogenomic and functional study with the ruminant pathogen *M. mycoides* subsp. *mycoides* (*Mmm*). We found that the extra copies of *atpA* and *atpD* belonged to a seven-gene cluster that had emerged during mycoplasma evolution and was spread by HGT. The proteins encoded by these genes were associated with a membrane ATPase activity. Based on our *in silico* analysis and experimental results, we propose a structural model of an F_1_-likeX_0_ ATPase specific to mycoplasmas.

## Materials and Methods

### Comparative genomics and phylogeny

Genome analysis and comparisons were carried out with MolliGen, a database for mollicute genomes (http://molligen.org) [12], and MBGD (http://mbgd.genome.ad.jp/) [13]. Protein sequences were named by their mnemonics (Table S2). GenBank IDs of the Type 3 ATPase proteins of *Mmm* are NP_975598.1 (MSC_0618), NP_975599.1 (MSC_0619), NP_975600.1 (MSC_0620), NP_975601.1 (MSC_0621), NP_975602.1 (MSC_0622), NP_975603.1 (MSC_0623) and NP_975604.1 (MSC_0624). Multiple alignments were generated with MAFFT [14] and MUSCLE [15] software. Unreliable sites were removed from the alignments with Gblocks [16] and Jalview [17]. Species and protein phylogenies were determined by the neighbour-joining (NJ), minimum evolution (ME) and maximum parsimony (MP) methods, in the MEGA4 package [18], and the maximum likelihood (ML) method in the Seaview [19] (integrating the phyML software [20]) or MEGA5 [21] packages. For trees constructed by the NJ, ME and MP methods, the “gap complete deletion” option was chosen and bootstrap analysis was carried out with 500 replicates. For phylogenetic reconstructions inferred from concatenated proteins α and β, the model best fitting the pattern of evolution, according to the Akaike information criterion (AIC), calculated with ProtTest [22], was LG+G+F. This model was used for ML phylogeny. The evolutionary divergence of sequences was estimated by the Poisson correction method in MEGA4 [18]. All positions containing gaps and missing data were eliminated from the dataset (complete deletion option). *In silico* predictions for operon structure were retrieved from the Database for prOkaryoticOperOns, (http://csbl1.bmb.uga.edu/OperonDB), [23]).

### Prediction of protein secondary structure and membrane topology

PSIPRED v.3.0 and pGenTHREADER were used to predict secondary structure and fold recognition, respectively. These programs are available from the PSIPRED Protein Structure Prediction Server (http://bioinf.cs.ucl.ac.uk/psipred/). The TMpred (http://www.ch.embnet.org/software/TMPRED_form.html) [24] and TMHMM (http://www.cbs.dtu.dk/services/TMHMM) [25] methods were combined for the prediction of transmembrane segments. SignalP-4.0 [26] was used to query signal peptide cleavages. The F_1_ and F_1_-like complex models of *Mmm* were drawn by similarity with the crystal structure of the *E. coli* F_1_-ATPase (Pdb id: 3oaa) [27] with the help of the Pymol software (http://www.pymol.org) [28]. The F_0_ subunits were drawn according to the ac_12_ and b_2_ models [29], [30]. The X_0_ proteins were schematized on the basis of their predicted 2D structures.

### Strains and culture conditions

The vaccine strain T1/44 (CIRAD-EMVT/PANVAC; Batch 002) of *Mmm* and the Δ619 mutant derived from it were grown at 37°C in modified Hayflick medium [31] supplemented with 20% horse serum, 0.4% pyruvate and 0.2% glucose. The growth of the T1/44 and mutant strains was monitored by measuring absorbance at 640 nm.

### Mutant selection and complementation

The *Mmm* Δ619 mutant was isolated from a library of *Mmm* T1/44 transformed with the plasposon PMT85/2res [32], by PCR screening with the specific probes MSC620.3 and MT85.1 (**Table S1**). A pure culture of the mutant was established after three cloning steps, by passage of the culture through filters with 0.2 µm pores. The purity of the mutant culture was checked by PCR, with the same primers used for the PCR screening assay (**Table S1**). A plasmid for the complementation of Δ619 was constructed as follows: the spiralin promoter was amplified by PCR with the PS1 and PS2 primers (**Table S1**), which include *Xba*I and *Xho*I sites. The amplified DNA was digested with *Xba*I and the resulting fragment was inserted into *Xba*I-linearized pMYSO1 [33] to generate pCC1. The genomic region corresponding to the *MSC_0619-MSC_0618* genes was amplified by PCR from T1/44 DNA with the MSC619.10 and MSC618.5 primers (**Table S1**). The PCR product was digested with *Xho*I and inserted into the *Xho*I site downstream from the spiralin gene promoter of pCC1. The resulting plasmid was named pCC2. T1/44 and Δ619 were transformed with 20 µg of pCC2 or pMYSO1, in the presence of polyethylene glycol, as described elsewhere [34].

### Protein extraction and preparation of membrane-enriched fractions

Total protein extracts were prepared from exponentially growing *Mmm* cells of the T1/44 and Δ619 strains. Cells were collected by centrifugation (12,000×*g* for 30 min, 4°C). The pellets were dispersed in phosphate-buffered saline (PBS, 139 mM NaCl, 1.9 mM KCl, 1.9 mM KH_2_PO_4_, 8 mM Na_2_HPO_4_,12 H_2_O pH 7.4) and washed three times in the same buffer. Cells were lysed by three one-minute cycles of sonication on ice with a microprobe (Vibra-Cell sonicator, Branson). For the isolation of membrane-enriched fractions, mycoplasma cells were washed three times in a washing buffer (50 mM Tris-HCl, 150 mM NaCl pH 7.4) and resuspended in lysis buffer (50 mM Tris HCl pH 7.4, 0.4 mM PMSF). The cells were lysed by three cycles of sonication for one minute on ice and the lysates were centrifuged for 10 min at 500×*g*. The supernatants were centrifuged for 1 h at 30,000×*g* (4°C). The pellets were dispersed in 50 mM Tris HCl pH 7.4 and centrifuged again for 1 h at 20,000×*g* (4°C). The final pellets were resuspended in 50 mM Tris HCl pH 7.4, frozen in liquid nitrogen and stored at −85°C until use. Protein concentration was determined with the DC reagent kit (BioRad), using bovine serum albumin as the standard. The total metabolic activity of the fractions was evaluated by measuring their capacity to generate H_2_O_2_ in response to the addition of glycerol. In T1/44, the transport of glycerol across the membrane and its phosphorylation during transport to generate glycerol-3-P leads to the production of H_2_O_2_, the formation of which is catalysed by the membrane-located L-α-glycerophosphate oxidase [35]. H_2_O_2_ was quantified as previously described [36].

### Generation of monospecific polyclonal antibodies

Peptide sequences for the *MSC_0618, MSC_0619, MSC_0620* and *MSC_0624* genes were retrieved from the *Mmm* genome sequence available from EMBL/GenBank/DDBJ entry BX293980. Transmembrane fragments were predicted with Phobius [37]. Protein fragments of 27 to 77 amino acids in length were selected on the basis of two criteria: (i) regions lying outside transmembrane fragments and (ii) regions with no UGA-encoded tryptophan residues. The corresponding gene regions were amplified by PCR from a genomic DNA template from *Mmm* strain T1/44 and inserted into pAff8c for the production of recombinant proteins in *Escherichia coli* strain BL21(DE3), these proteins then being purified as previously described [38]. The purity of the recombinant proteins, as assessed by SDS-PAGE and Coomassie blue staining, exceeded 80%. Quality control was also carried out for the proteins, by electrospray ionization mass spectrometry, before the removal of an aliquot of 500 µg of the recombinant protein for rabbit immunization. Serum samples were obtained and subjected to affinity purification according to national guidelines (Swedish permit 84-02) and standard procedures [39]. The affinity purification process was used to remove non-specific antibodies and antibodies binding to the fusion tag. The specificities of the monospecific polyclonal antibodies were assessed by dot and western blotting before use. Three antibodies – MSC618002, MSC619001, and MSC620002 – were finally selected. The rabbit polyclonal serum against N-ter LppQ (anti-LppQ) was a gift from Prof. J. Frey [40].

### Protein electrophoresis, immunoblotting and mass spectrometry

Proteins were separated by electrophoresis, as previously described [41], in 10% polyacrylamide gels. For protein immunodetection, ∼10 µg of protein from T1/44 or Δ619 was loaded onto the gel. Proteins were electroblotted onto a nitrocellulose membrane in a semidry transfer unit (Trans-Blot® SD semi-dry electrophoretic transfer cell, BioRad) and probed with the polyclonal antibodies MSC618002, MSC619001, MSC620002 and Anti-LppQ, followed by horseradish peroxidase-conjugated secondary antibodies (Sigma). The Supersignal® West Pico Chemiluminescent Substrate kit (WestPicoLuminol, Pierce) was used for detection. For the identification of proteins in membranes by LC-MS/MS, membrane-enriched fractions were separated by electrophoresis in 10% polyacrylamide gels. Coomassie blue G250 staining was used to visualize the protein bands. The gel was sliced into 16 sections, which were subjected to trypsin (Sigma) digestion. The resulting peptides were further analysed by online capillary liquid chromatography (LC Packings) coupled to an MS/MS orbitrap mass spectrometer (ThermoFinnigan). Peptides were separated on a 75 µm (internal diameter)×15 cm C18 PepMap column (LC Packings), with a flow rate of 200 nl/min. Peptides were eluted with a 5–40% linear gradient of solvent B over a period of 35 min (solvent A was 0.1% formic acid in 5% acetonitrile, and solvent B was 0.1% formic acid in 80% acetonitrile). The mass spectrometer was operated in positive-ion mode, at a needle voltage of 2.2 V and a capillary voltage of 49 V. Data were acquired in a data-dependent mode consisting of full-scan MS over the range m/z 300–1700 or five full-scan MS/MS for the five most intense ions in the preceding MS spectra. MS/MS data were acquired with a 2 *m/z*-unit ion isolation window and relative collision energy of 35%. Peptides were identified with SEQUEST, through the Bioworks 3.2 interface (Thermo-Finnigan), using a database containing both direct and antisense sequences for *Mmm* coding sequence (CDS) and intergenic sequence data.

### ATPase activity assays

ATPase assays were performed with EnzChek (Invitrogen), in the presence of 80 µM ATP and membrane-enriched fractions (60 µg protein), at room temperature. Absorbance at 360 nm was measured every 90 s, for 40–50 min, following the addition of ATP. A control assay lacking the membrane-enriched fraction was performed for the deduction of background ATP-self-hydrolysis values. Absorbance was converted into the amount of free orthophosphate (Pi) released, by comparison with standard curves for Pi (0.01 mM to 0.12 mM KH_2_PO_4_). The statistical *t-student* test at 0.05 significance level was applied to compare the Pi released with the membrane-enriched fraction from the T1/44 and Δ619strains.

### RT-PCR analysis

Total RNA was extracted with Trizol from exponentially growing T1/44 and Δ619 cells subjected to DNase I treatment. We then synthesized cDNA from 500 ng of total RNA, with the SuperScript II reverse transcriptase (Invitrogen) and gene-specific primers, according to the manufacturer's instructions. A control lacking the reverse transcriptase was also included for each sample and was processed in tandem. For the amplification of intergenic regions, we used the following cycling parameters: 95°C for 5 min, followed by 35 cycles of 95°C for 40 s, 54°C for 40 s and 72°C for 40 s, with a final extension at 72°C for 7 min. The primers used are described in **Table S1**. The quality of the RNA was checked with the MSC679.1 and MSC679.2 primers for amplification of the *gap* gene. The amplified products were run on a 1% agarose gel and visualized with ethidium bromide staining.

### Limited proteolysis assays

Exponentially growing *Mmm* cells were collected by centrifugation, washed with PBS pH 7.5 and dispersed in 200 µl of PBS pH 8.4. Limited proteolysis was performed on intact cells or on cells disrupted by brief sonication. One volume of agarose beads coated with trypsin (Promega) was added to 2.5 volumes of suspended cells. Samples were incubated at 37°C for 6 hours. Trypsin was removed by centrifugation and protein profiles were analysed by western blotting with MSC618002, MSC620002 and Anti-LppQ polyclonal antibodies.

## Results

### Extra copies of genes related to F_1_F_0_ ATPase subunits alpha and beta are found in mycoplasma genomes

**Figure 2:**
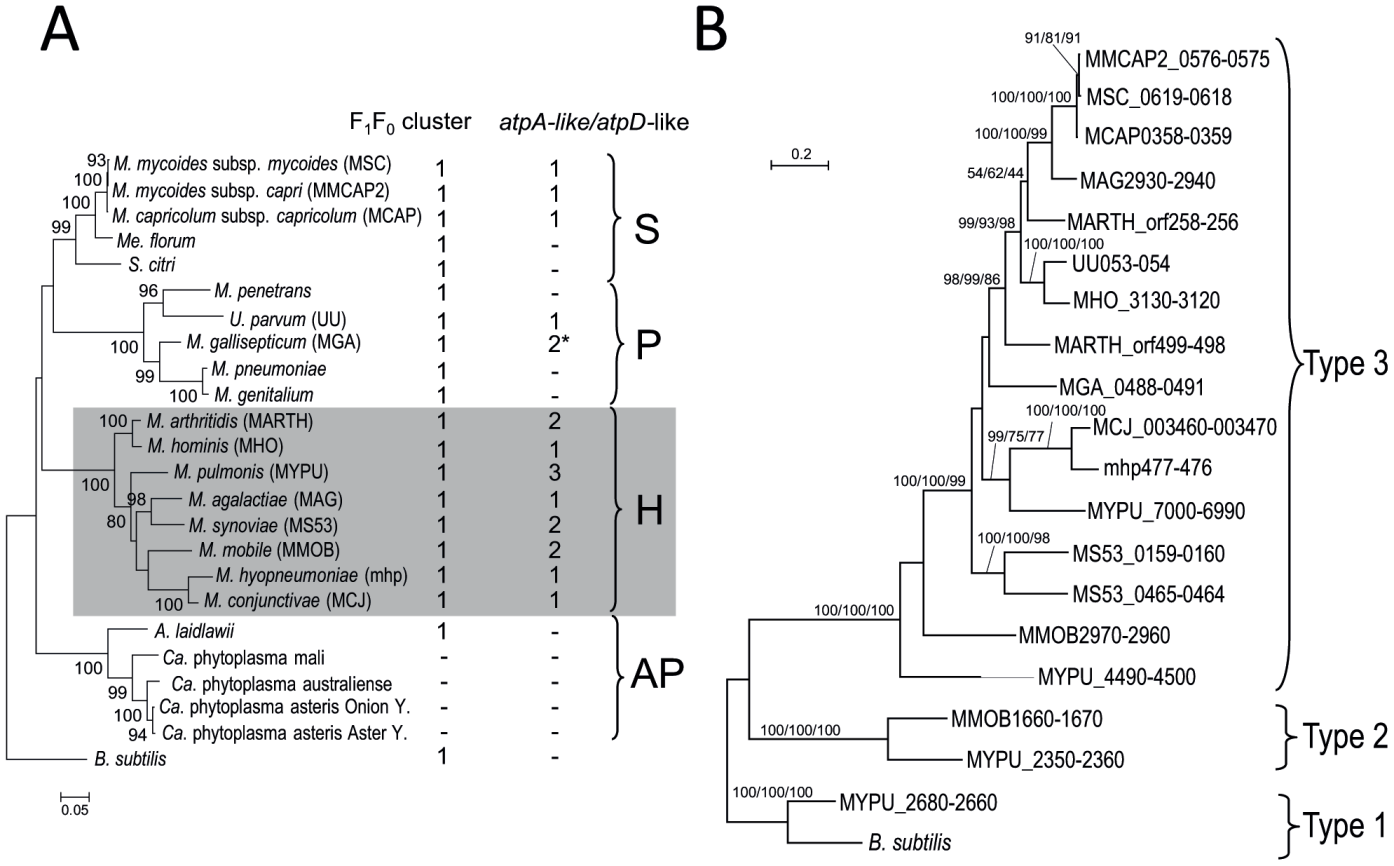
Distribution and evolution of extra copies of *atpA*-like and *atpD*-like genes in mollicutes. **A**. The number of typical F_1_F_0_ ATPase operons and of extra copies of *atpA*-like/*atpD*-like pairs of genes are indicated for each species. * In *M. gallisepticum*, one of the two extra copies only contains a truncated *atpD*-like gene. The 16S rDNA phylogenetic tree was generated by the ML method; bootstrap values of more than 50% are indicated. *Bacillus subtilis* was chosen as an outgroup species. Phylogenetic groups are indicated: S, Spiroplasma; H, Hominis; P, Pneumoniae; AP, Acholeplasma/Phytoplasma. Mnemonic codes are indicated in brackets besides species names when useful. **B**. The amino acid sequences of the proteins encoded by the *atpA*-like and *atpD*-like genes were concatenated and a multiple alignment was generated. Protein sequences of Type 1 *atpA* and *atpD* genes from *M. pulmonis* and *B. subtilis* (GenBank ID: *atpA*, NP_391564.1; *atpD*, NP_391562.1) were used as outgroup. The multiple sequence alignment was curated with GBLOCK to remove unreliable sites and a final round of manual editing was performed with Jalview. Phylogenetic trees were generated by ML, NJ and MP methods. The tree represented was obtained by the ML method. The aLRT/Bootstrap values corresponding to these three methods are indicated on the branches, in the following order: ML/NJ/MP. Sequences are labelled by their mnemonics, see also **Table S2** for details.

Clusters of eight genes encoding the typical F_1_F_0_ ATPase have been identified in all mollicutes with the exception of plant pathogenic phytoplasmas. Extra copies of *atpA* (subunit α, F_1_-sector) and *atpD* (subunit β, F_1_-sector) genes, organized in pairs, have also been annotated in several mycoplasma genomes. Blastp queries have identified such extra copies in many species (**Figure 2A**). In the Hominis phylogenetic group which includes mycoplasmas pathogenic to humans and various animals, all eight of the available genomes contain one to three extra copies of the *atpA*-like/*atpD*-like gene pair. In the Pneumoniae group which also contains human and animal pathogens, two of the five species had extra copies. One extra pair of genes was found in the genome of the human urogenital pathogen *Ureaplasma parvum* (serovar 3 strain ATCC 700970) and in the other two complete *Ureaplasma* genomes available (serovar 3 strain ATCC 27815 and serovar 10 strain ATCC 33699, not shown). In the genome of the bird pathogen *Mycoplasma gallisepticum*, one pair of *atpA*-like/*atpD*-like genes was found. An additional gene related to *atpD* was also detected at another locus on the chromosome. Within the Spiroplasma phylogenetic group, an extra pair of genes was found in the genomes of the ruminant pathogens *Mmm, Mycoplasma mycoides* subsp. *capri* and *Mycoplasma capricolum* subsp. *capricolum*. By contrast, no extra copies of genes related to the F_1_F_0_ ATPase were predicted in the genomes of *Mesoplasma florum* (isolated from the surface of a lemon tree flower) and *Spiroplasma citri* (plant pathogen transmitted by insects). All the proteins encoded by the additional *atpA*-like and *atpD*-like genes were predicted to contain the corresponding Prosite motif (PS00152 ATPASE ALPHA BETA) and most were annotated accordingly.

**Figure 3:**
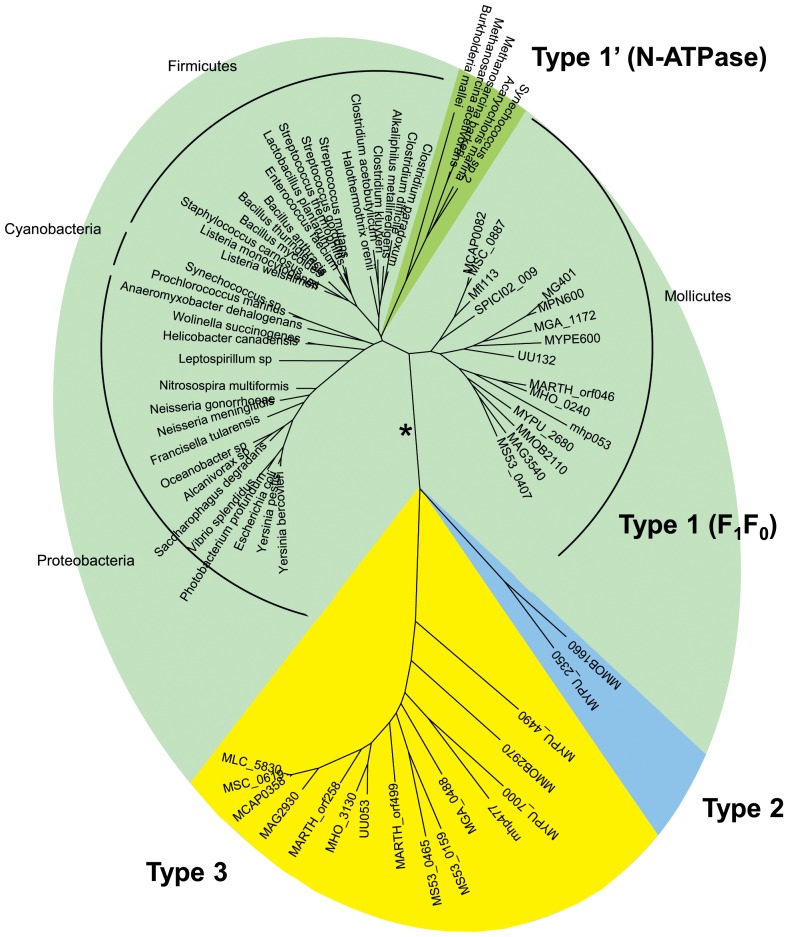
Evolution of *atpA* and *atpA*-like genes in bacteria. The phylogenetic tree was inferred from the amino acid sequences of ATPase alpha subunits encoded by *atpA* and *atpA*-like genes. Multiple alignment was generated with MUSCLE. The phylogenetic tree was generated by the ML method. Branches corresponding to Type 1, Type 1′, Type 2 and Type 3 proteins were supported by 96–100% bootstrap values. The ML, NJ, MP and ME methods generated trees with similar topologies, except alternative branching of N-ATPases using NJ or ME (indicated by a star). Main bacterial groups are indicated. Proteins from mollicutes are named by their mnemonics, others by the species name. See **Table S2** for details.

We characterized the evolutionary relationship between the typical F_1_F_0_ ATPase and these extra copies of the *atpA* and *atpD* genes, by generating multiple alignments of the corresponding α-like and β-like protein sequences and inferring phylogenetic trees. A selection of F_1_F_0_ ATPase homologs from non mollicute bacteria and some related N-ATPases [11] were included in this analysis. The general shape of the tree was identical for both proteins (the tree obtained for α and α-like proteins is shown in **Figure 3**), indicating a clear divergence of the mycoplasma-specific extra copies from both genuine bacterial F_1_F_0_ ATPase homologs and N-ATPase homologs. Indeed, the typical F_1_F_0_ ATPase subunits from mollicutes clustered with the orthologous proteins found in most bacteria, in a sub-tree with a topology resembling that for 16S rDNA. These typical F_1_F_0_ ATPase copies and atypical N-ATPases are referred to hereafter as Type 1 and Type 1′, respectively.

The additional copies of α-like and β-like subunits from mollicutes clustered into two phylogenetically remote branches, identified here as Type 2 and Type 3. Type 2 copies were found only in two distantly related species from the Hominis group: the rodent pathogen *Mycoplasma pulmonis* [42] and *Mycoplasma mobile* [43], which was isolated from a fish. Both these species also had Type 3 genes clustered with all the other extra copies, in a monophyletic group. The separation of the Types 1, 1′, 2 and 3 copies was strongly supported by an approximate likelihood-ratio test.

Thus, many mycoplasma genomes contain one or several extra copies of genes related to *atpA* and *atpD* and α-like and β-like subunits they encode diverge markedly from the typical F_1_F_0_ ATPase subunits found in mycoplasmas and most bacteria.

### Type 3 clusters have been exchanged in the three documented HGT between mycoplasmas

A phylogenomic study of Type 2 and Type 3 gene pairs was conducted with concatenated α-like and β-like proteins (**Figure 2B**), to clarify the evolutionary relationships. As expected, Type 2 and Type 3 pairs of proteins clustered into two well-supported branches, indicative of their distant relationship. The topology of the Type 3 subtree was only partially consistent with the species tree inferred from 16S rDNA (see **Figure 2A**). First, in some species (*Mycoplasma arthritidis*, *M. pulmonis*) pairs of homologous proteins were not found close together, as would be expected for recently duplicated genes. Second, the proteins were not distributed along the well-defined main phylogenetic branches for mollicutes. Proteins from *Mycoplasma agalactiae* (Hominis phylogenetic group) and species of the so-called mycoides cluster (*Mmm*, *M. mycoides* subsp. *capri* and *M. capricolum* subsp. *capricolum*, Spiroplasma phylogenetic group) were found to be closely related. This finding is consistent with published reports of HGT between ruminant pathogens [6]. Similarly, proteins from the urogenital species *Mycoplasma hominis* (Hominis group) and *U. parvum* (Pneumoniae group) were positioned on sister branches, consistent with hypothetical gene exchange between these species [3]. The complete pair of genes found in the bird pathogen *M. gallisepticum* (MGA_0488/MGA_0491) was not clearly related to any other cluster, but was located at some distance from *U. parvum* proteins in the tree, despite the common ancestry of these species. Moreover, trees including the protein encoded by the isolated *atpD*-like gene of *M. gallisepticum* (MGA_1321d) showed that this protein was strongly related to the homologous protein from *Mycoplasma synoviae*, consistent with HGT (data not shown).

**Figure 4:**
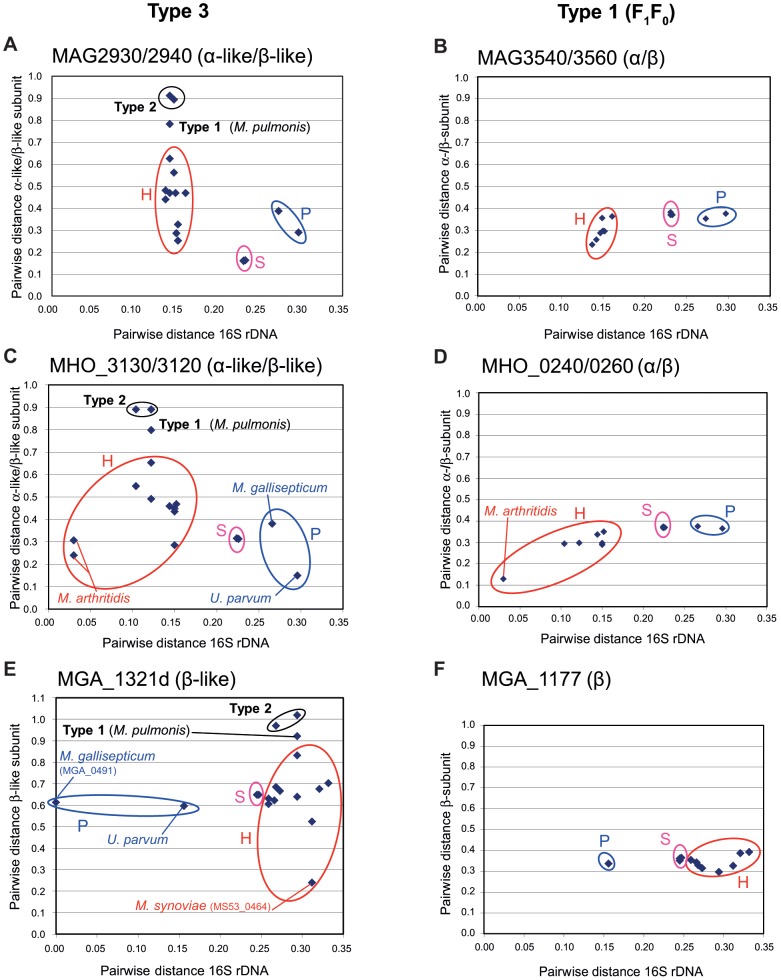
Evolutionary distance between clusters. Amino acid sequences of genes encoding α-like and β-like proteins of the Type 2 and Type 3 clusters (panels A, C and E) and α- and β-subunits of F_1_F_0_ Type 1 clusters (panels B, D and F) were concatenated and multiple alignments were generated. Multiple sequence alignments were curated with GBLOCK to remove unreliable sites and a final round of manual editing was performed with Jalview. Evolutionary distances were calculated with Type 3 pairs thought to have been exchanged through HGT as references. These distances are shown as a function of the evolutionary distance between species inferred from 16S rDNA data. The Type 3 pairs concerned were MAG2930/2940 (*M. agalactiae*), MHO_3130/3120 (*M. hominis*) and MGA_1321d (*M. gallisepticum*). In the last case, the analysis was based exclusively on the truncated *atpD*-like gene. Homologs from a phylogenetic group are circled: H, Hominis; P, Pneumoniae; S, Spiroplasma.

We estimated the degree of relatedness between Type 3 α-like/β-like pairs of proteins, by calculating pairwise distances between homologous pairs, using *M. agalactiae*, *M. hominis* and *M. gallisepticum* as references (**Figure 4** **, panels A, C and E**). For the purpose of comparison, pairwise distances were also calculated between concatenated α/β pairs from the Type 1 F_1_F_0_ ATPase (**Figure 4** **, panels B, D and F**). The F_1_F_0_ ATPase pairwise distances were roughly related to the evolutionary distance estimated on the basis of 16S rDNA pairwise distances, but no such correlation was observed for Type 3 proteins. In all cases, the minimal pairwise distances were recorded for homologs for which HGT was predicted. The most remarkable illustration of this finding concerns the isolated *M. gallisepticum* β-like copy (MGA_1321d), for which calculated pairwise distance from *M. synoviae* homolog MS53_0464 was only 0.239 whereas the other β-like copy (MGA_0491) present in *M. gallisepticum* was much more distant (pairwise distance: 0.613) (**Figure 4E**). Two additional features emerged from these graphs. First, both Type 1 and Type 2 homologs presented high pairwise distances from the Type 3 references, with the highest values obtained for Type 2 homologs. This suggests that Type 2 and Type 3 gene pairs have diverged considerably since their evolutionary separation. Second, the range of pairwise distances between homologs from the Hominis group was higher for Type 3 pairs than for F_1_F_0_ ATPase pairs. For instance, when *M. agalactiae* proteins were used as references, pairwise distances ranged from 0.252 to 0.625 (Δ = 0.373) for Type 3 (**Figure 4A**) but only from 0.234 to 0.363 (Δ = 0.129) for Type 1 (**Figure 4B**). Thus, even within the Hominis phylogenetic group, the level of divergence between Type 3 pairs was high, suggesting a complex evolutionary history marked by HGT and/or bursts of evolution.

### Five genes colocalize with extra *atpA*-like and *atpD*-like copies

**Figure 5:**
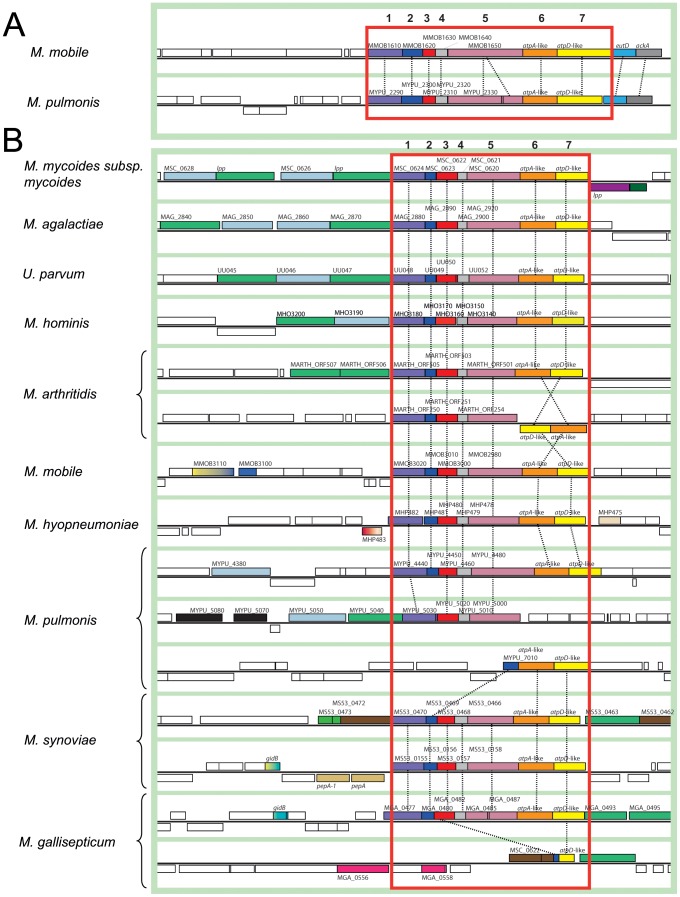
Genomic contexts of Type 2 and Type 3 clusters in mycoplasmas. Homologous genes are indicated by boxes of the same colour, connected by dashed lines. The genomic regions containing the Type 2 (A) and Type 3 (B) clusters are framed in red. The schematic diagram was generated from screenshots obtained from the MBGD database. Mnemonics and gene names are indicated; genes from the clusters are numbered arbitrarily from 1 to 7. The genome structures of *M. mycoides* subsp. *capri* and *M. capricolum* subsp. *capricolum* were identical to that of *Mmm*.

We analysed the genomic context of Type 2 and Type 3 *atpA*-like and *atpD*-like genes. A genomic region surrounding Type 2 *atpA*-like/*atpD*-like genes and including nine syntenic genes was common to *M. mobile* and *M. pulmonis* (**Figure 5A**). Two genes located downstream from the *atpA*-like/*atpD*-like genes encoded a phosphate acetyltransferase (*eutD*) and an acetate kinase (*ack*). These tandemly associated genes were found at various locations on the chromosomes of most mycoplasmas, whether or not *atpA*-like/*atpD*-like genes were present. By contrast, the five genes upstream from the *atp*A-like/*atpD*-like genes encoded conserved hypothetical proteins. None presented homologs that could be identified by blastp search in other mollicutes or any other organism. These genes are listed in **Table S2**. For convenience, the seven genes of the cluster were arbitrarily numbered 1 to 7. Gene 5 of the Type 2 cluster in strain UABCTIP of *M. pulmonis* was interrupted by a stop codon [42], as confirmed by PCR and sequencing. However, this mutation was not found in other three strains of *M. pulmonis* (not shown).

Five genes encoding hypothetical proteins were also conserved upstream from most Type 3 *atpA*-like/*atpD*-like genes (**Figure 5B** **, Table S2**). Blastp queries retrieved no putative homologs for the proteins encoded by these five genes. In several species, these clusters of genes were preceded by genes encoding lipoproteins, transmembrane proteins and hypothetical proteins. Phylogenetic reconstructions suggested that genes 1 to 5 and some of these upstream genes may have been exchanged, together with the *atpA*-like/*atpD*-like genes, during HGT. Complete Type 3 clusters including seven non disrupted genes were found in eleven of the twelve species in which Type 3 *atpA*-like/*atpD*-like genes were identified. The only exception was *M. gallisepticum* (strain R-low), the genome of which contained a seven-gene cluster in which gene 5 presented a non-sense mutation that was checked by PCR and sequencing. This mutation was also found in the recently sequenced genome of a derived, non-pathogenic strain after a large number of passages (strain R-High), but vaccine strain F had an intact cluster indicating the recent nature of the mutation [44]. All sequenced *M. gallisepticum* genomes also contained a degraded cluster with only partial sequences of genes 1 and 7. Remarkably, the genes located on either side of the degraded cluster were found to be closely related to genes flanking one of the *M. synoviae* clusters (**Figure S1**). This observation is consistent with the hypothesis of HGT between bird mycoplasmas. Additional Type 3 clusters presumably split by genome re-organization events were found in *M. arthritidis* and *M. pulmonis* (**Figure 5B**). In particular, the gene encoding Protein 2 in *M. pulmonis* was missing from the first part of the cluster but was found next to the *atpA*-like/*atpD*-like genes, suggesting that this cluster may have been reshuffled by several successive events. Only *M. synoviae* harboured two complete Type 3 clusters.

In summary, both Type 2 and Type 3 *atpA*-like/*atpD*-like pairs of genes were found clustered with five genes encoding proteins of unknown functions with no obvious similarities to any other proteins from databases. These clusters of seven genes were found in all the species from the Hominis group, consistent with a common ancestry. Horizontal gene transfer may account for the presence of Type 3 clusters in species of the Pneumoniae and Spiroplasma phylogenetic groups.

### Type 2 and Type 3 clusters: indications for an F_1_-likeX_0_ structure

**Figure 6:**
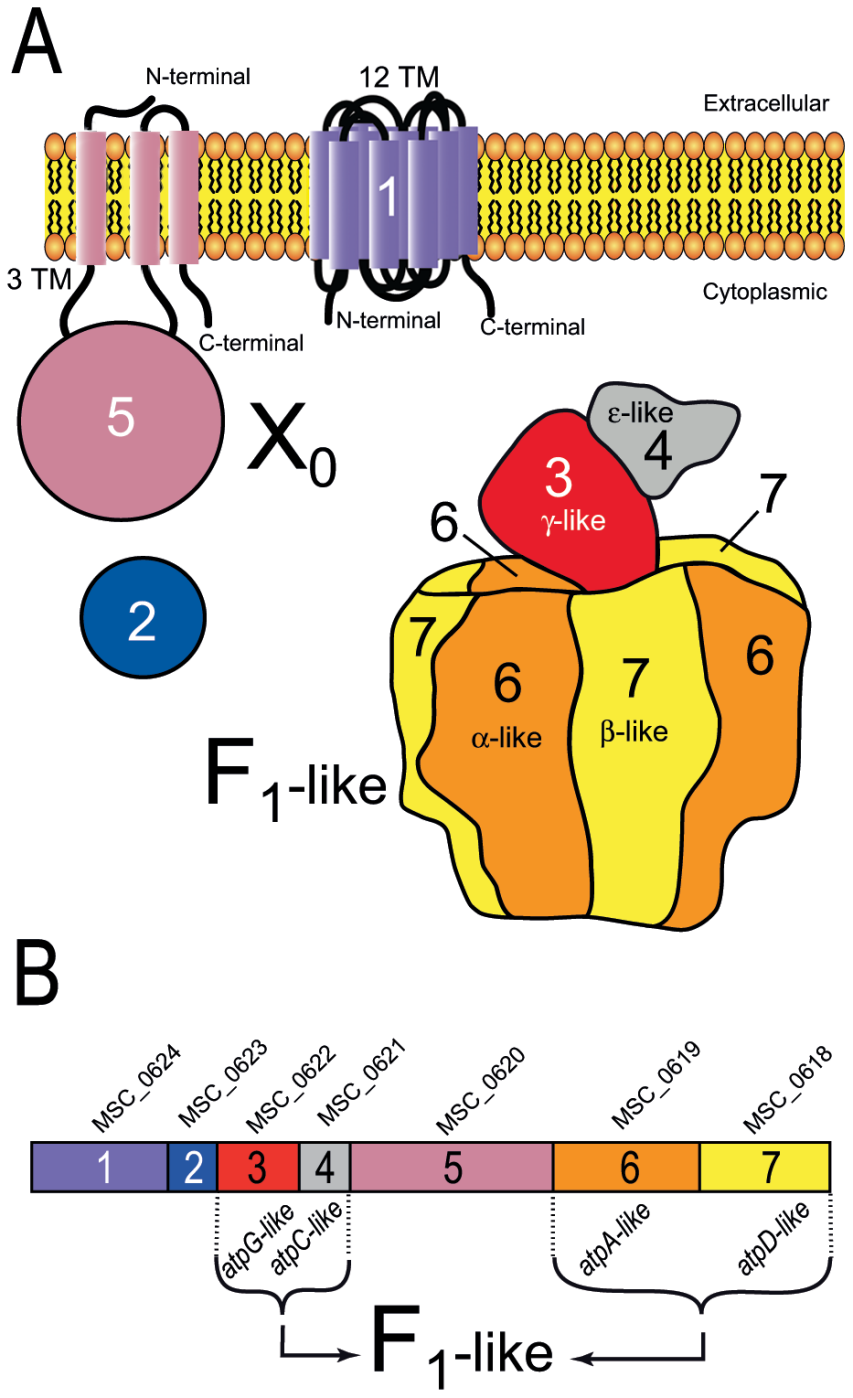
Model of an F_1_-likeX_0_ ATPase encoded by the seven-gene clusters of Types 2 and 3 specific to mycoplasmas. **A**. The F_1_-like complex model of *Mmm* was drawn by similarity with the crystal structure of the *E. coli* F_1_-ATPase (Pdb id: 3oaa) [27] with the help of the Pymol software (http://www.pymol.org) [28]. The X_0_ complex proteins of *Mmm* were schematized on the basis of 2D structure predictions. Proteins 1 and 5 are depicted associated with the membrane, in accordance with the predicted transmembrane segments. Based on *in silico* and experimental results, the F_1_-like complex, Protein 2 and the main part of Protein 5 were predicted to be cytoplasmic. Within this model, the F_1_-like and the X_0_ sectors are represented, but the way they could interact remains largely unclear. **B**. The genes of the clusters were arbitrarily numbered from 1 to 7. Gene names are indicated above the boxes representing the genes. TM, transmembrane segments. The proteins encoded by genes 3, 4, 6 and 7 were found to be related to the subunits γ, ε, α and β of the F_1_F_0_ ATPase, respectively.

The Type 2 and Type 3 gene clusters did not display strong sequence similarity, but they were organized similarly and proteins encoded by genes 1, 2, 3 and 4 were of the similar lengths in the two types (**Figures 6B** **and S2**). We investigated the function of Type 2 and Type 3 clusters further, by analysing and comparing the predicted proteins. This analysis is illustrated in **Figure S3** for proteins 1–7 from *Mmm*.

In accordance with the *atpA*-like and *atpD*-like annotation found in most mycoplasma genomes, the primary and secondary structures of Proteins 6 and 7 displayed significant similarity to those of the α- and β-subunits of bacteria F_1_F_0_ ATPases, respectively. The presence of Walker consensus sequences A and B suggested that these proteins were able to bind nucleotides. The Walker A motif, also named P loop, interacts with the ATP phosphate groups while the Walker B motif is involved in ATP hydrolysis [45]. Protein 6 possessed the DELSEED-loop signature of the β-subunit, which is essential for the ε-subunit to play a role as an inhibitor [27], [46]. Protein 3 (∼36 kDa) displayed less than 22% and 14% amino-acid identity to γ-subunits of mycoplasma and other bacteria F_1_F_0_ ATPases, respectively. However, the predicted secondary structure included long helices at the N- and C-terminal ends, surrounding a mixed α-helix/β-strand domain (**Figure S3C**). Fold recognition software predicted a γ-subunit F_1_ ATPase with medium confidence (score = 41). Protein 4 was predicted to have eight strands at the N-terminal end and two helices at the C-terminal end (**Figure S3D**). Despite a very low level of sequence similarity to the ε-subunit of bacteria F_1_ ATPases (less than 11% similarity), the predicted secondary structure and the topology of Proteins 4 resembled the known structure of the ε-subunit of bacteria F_1_ ATPases. An ε-subunit fold was therefore predicted with a low level of confidence (score = 31).

Protein 7 (β-like) in cluster 2 (*M. mobile* and *M. pulmonis*) had an extension of about ∼30 kDa at the N-terminus. The resulting additional mass of ∼90 kDa located on top of the potential hetero-hexamer (αβ)_3_ might affect its connection with the peripheral stalk subunit. Moreover, no ORFs in clusters 2 and 3 were found to code the δ-subunit or b-subunit homologs that are essential for the formation of the peripheral stalk of the F_1_F_0_ ATPase. Without a peripheral stalk to anchor (αβ)_3_ to the membrane, the enzyme is likely to function as an ATPase rather than an ATP synthase. Together, Proteins 6, 7, 3 and 4 from both Type 2 and Type 3 could assemble into a functional complete F_1_-like ATPase (**Figure 6A**).

No sequence similarity was found between Proteins 2 and proteins of known function or structure. No transmembrane segment or signal peptide was predicted suggesting that Protein 2 was cytosolic and might therefore interact with the F_1_-like ATPase. Short (19 kDa) and long (35 kDa) versions of Protein 2 were found in Types 2 and 3, respectively. Based on molecular weight alone, the shorter version of the protein could be a homolog to the 19 kDa δ-subunit. However, its predicted secondary structure (**Figure S3B**) was clearly different from that of the δ-subunit which is an α-helix rich- protein.

According to TMHMM and TMPred predictions, Protein 1 (∼55 kDa) contained twelve transmembrane helices connected by short loops (**Figure S3A**) and both the N- and C-terminal ends were located on the cytoplasmic side of the membrane. Protein 5 was predicted to be anchored in the membrane through at least two transmembrane helices linked by a short loop near the C-terminus (amino acids 707–768 for MSC_0620 from *Mmm*, **Figure S3E**). The predicted secondary structure included N-terminal (amino acids 1–190) and C-terminal (aa 460–770) regions rich in helices and a central region (aa 190–460) containing both helices and strands. A cleavable (between positions 24 and 25) signal peptide mediating membrane targeting was predicted by SignalP-4.0 software. Finally, two models for the topology for Protein 5 were predicted depending on the program used. According to TMHMM predictions, Protein 5 is probably mostly exposed at the cell surface (aa 31–709), whereas TMpred predicted it to be mainly cytoplasmic using (**Figure S3F**). Fold recognition with pGenTHREADER software predicted no meaningful topology for Proteins 1 and 5. All the membrane-spanning segments of Proteins 1 and 5 were highly hydrophobic, with no charged residues as expected for rotor and stator F_0_ subunits. However, Proteins 1, 2 and 5 were clearly not homologous to any known F_0_ subunits of ATPases.

### Experimental study of the Type 3 cluster in *Mmm*: evidence for membrane ATPase activity

We investigated whether the seven genes of the clusters related to F_1_ ATPase were actively expressed and whether they displayed any associated ATPase activity, by carrying out an experimental study on the Type 3 cluster of *Mmm*. This particular mycoplasma species was chosen because: (i) it is predicted to contain a single, apparently complete Type 3 cluster, (ii) *Mmm* is a major pathogen of ruminants and its biology and pathogenicity remain poorly understood, (iii) *Mmm* grows more rapidly than other mycoplasma species and several genetic tools have been developed for this species, including replicative *oriC* plasmids and transposon-based mutagenesis methods [47]. Proteins 1–7 of *Mmm* were named by their mnemonics MSC_0624-MSC_0618 (**Table S2**), with MSC_0618 and MSC_0619 corresponding to the β-like and α-like subunits, respectively.

### The Type 3 cluster forms an operon in *Mmm*

In prokaryotic genomes, functionally related genes are frequently organized into operons. *In silico* predictions for operon structure were retrieved from the DOOR database (Database for prokaryotic operons, http://csbl1.bmb.uga.edu/OperonDB, [23]). The seven genes of *Mmm* Type 3 cluster were predicted to be organized into a single operon on the basis of several features including synteny and intergenic regions which were less than 6 nt in length. An operon structure was also predicted for all eight mycoplasma Type 3 clusters for which information was available from the DOOR database at the time of analysis. Interestingly, in both *Mmm* and other mollicutes (*M. pulmonis, M. synoviae, U. parvum, M. agalactiae, M. capricolum* subsp. *capricolum, M. gallisepticum*), the operon was predicted to contain several additional genes. Almost all these additional genes encoded proteins annotated as (conserved) hypothetical proteins or lipoproteins, with the exception of a glycyl-tRNA synthetase-encoding gene in *M. pulmonis*. In *Mmm*, the predicted operon contained the putative lipoprotein-encoding gene *MSC_0625* and *MSC_0626*, encoding a predicted transmembrane protein, in addition to the Type 3 genes *MSC_0618-MSC_0624*. The actual sensitivity and specificity of the DOOR predictor for mycoplasma genomes remain unknown because no global experimental operon data are available for Type 3- and Type 2-containing genomes. However, only 10 to 18 operons of seven or more genes were predicted in all the genomes considered. This suggests that the prediction of operons is highly specific in these mycoplasma genomes.

**Figure 7:**
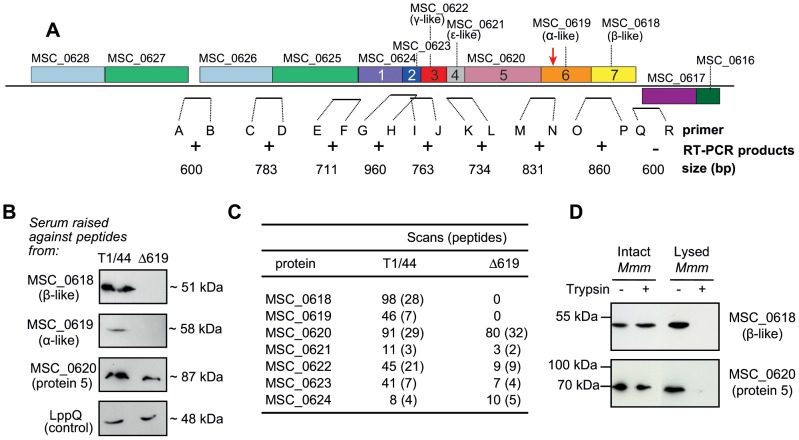
Operon structure and expression of the genes of the Type 3 cluster in *Mmm*. **A**. RT-PCR experiments were carried out on intergenic regions to demonstrate the co-transcription of the *MSC_0618* to *MSC_0627* genes. The region of the genome region surrounding the Type 3 cluster in *Mmm* is shown. Gene mnemonics and numbers are shown for the Type 3 cluster. The site of transposon insertion in the *MSC_0619* disrupted mutant (Δ619) is indicated by an arrow. Expected sizes of the putative transcripts are indicated. Amplification products of the expected sizes were obtained with primers binding within and upstream from the cluster (+) but not downstream from the cluster (−). **B**. Immunodetection of proteins from the Type 3 cluster of T1/44 and Δ619. A control for membrane protein detection was included, in the form of an antibody against a membrane protein, LppQ (anti-LppQ serum kindly supplied by Prof. J. Frey). **C**. Nano-LC-MS/MS detection of Type 3 ATPase proteins. Numbers of scans and distinct peptides detected are indicated. **D**. Evaluation of the sensitivity of Protein 5 and β-like subunit to trypsin degradation. Intact and lysed cells of *Mmm* T1/44 were incubated with (+) or without (−) trypsin enzyme coated on beads for six hours. Protection against hydrolysis was assessed by immunodetection with antibodies raised against Protein 5 (MSC_0620) and β-like subunit (MSC_0618).

Standard RT-PCR assays were then undertaken to demonstrate the production of transcripts overlapping the various CDS regions within the cluster in *Mmm*. Based on the DOOR predictions, we carried out nine RT-PCR assays with the primers listed in **Table S1**. An overview of the binding sites of the primers is provided in **Figure 7A**. Products of the expected size were obtained for eight out of nine RT-PCRs, confirming linked transcription of the open reading frames in the Type 3 gene cluster in *Mmm*. No product was detected when the *MSC_0617-MSC_0618* intergenic region was amplified. These results suggest that the genes of the Type 3 cluster and at least three upstream genes constitute an operon expressed in *Mmm*.

### Protein production and localization in *Mmm*

We assessed the production of membrane-associated proteins from axenic cultures of *Mmm* grown in pyruvate-supplemented Hayflick culture medium. Membrane-enriched extracts were subjected to SDS-PAGE and western-blotting with polyclonal antibodies raised against synthetic peptides designed from the protein sequences of MSC_0618, MSC_0619 and MSC_0620. Each of the three polyclonal antibodies detected a single band corresponding to a protein of the expected size in the membrane protein extracts of *Mmm* (**Figure 7B**). The same western-blotting pattern was observed whatever the initial optical density at 640 nm of the mycoplasma culture batch (data not shown). These data suggest that MSC_0618-MSC_0620 proteins were constitutively produced by the mycoplasma cells in the culture conditions tested. All nine MSC_0618-MSC_0626 proteins were unambiguously identified by a proteomic approach based on nano-LC-MS/MS (**Figure 7C**) from the set of extrinsic and integral proteins detected in membrane protein extracts. Thus, *Mmm* produced the nine proteins of the cluster and all nine proteins remained associated with the membrane under the experimental conditions used to isolate membrane-enriched fractions.

As indicated above (**Figure S3F**), a cytoplasmic location was unambiguously predicted for five proteins, including the F_1_-like subunits MSC_0618 and MSC_0619. The other two proteins, MSC_0620 and MSC_0624, were predicted to contain transmembrane segments. The TMHMM and TMPred programs predicted twelve transmembrane segments with the N-terminus located in the cytoplasm for MSC_0624. For MSC_0620, no unambiguous topology model could be obtained with the prediction methods used. Indeed, TMHMM data suggested that amino-acid residues 31 to 709 were exposed at the surface, whereas residues 31–707 were predicted to be cytoplasmic by TMpred. We obtained experimental data for the orientation and topology of the putative ATPase complex, the membrane topology of MSC_0620 and the localization of the F_1_-like component MSC_0618, by carrying out limited proteolysis of *Mmm* cells with trypsin immobilized on agarose beads. The accessibility of proteins to trypsin digestion was assessed in intact cells and in cells disrupted by mild sonication. The proteolysis of MSC_0618 and MSC_0620 following the addition of trypsin was evaluated by western-blotting with the monospecific polyclonal antibodies MSC618002 and MSC620002, respectively. Under identical experimental conditions, no hydrolysis of MSC_0618 was observed after six hours (**Figure 7D**). By contrast, disruption of the cells before the addition of trypsin resulted in the nearly complete digestion of MSC_0618, suggesting that an intact plasma membrane was required to protect MSC_0618 against digestion. Thus, MSC_0618 (β-like subunit) is located in the cytoplasm, providing strong evidence in favour of a cytoplasmic location for the whole F_1_-like structure.

After treatment with trypsin for six hours, MSC_0620 digestion was observed in disrupted cells, but not in intact cells. As the polyclonal antibodies used in proteolysis experiments were raised against a polypeptide corresponding to amino acids 182–231, the trypsin accessibility assay indicates that this fragment was on the cytoplasmic side of the membrane. In the simplest topology model consistent with the experimental data, MSC_0620 is a protein exposed mostly on the cytoplasmic side of the membrane and possessing a membrane-anchoring domain consisting of two transmembrane segments at its C-terminal end. In this model, the orientation of MSC_0620 may allow it to interact with both the membrane and the F_1_-like structure of the complex.

### An *Mmm* Δ619 mutant displays low levels of ATPase activity: the cluster is associated with ATPase activity

**Figure 8:**
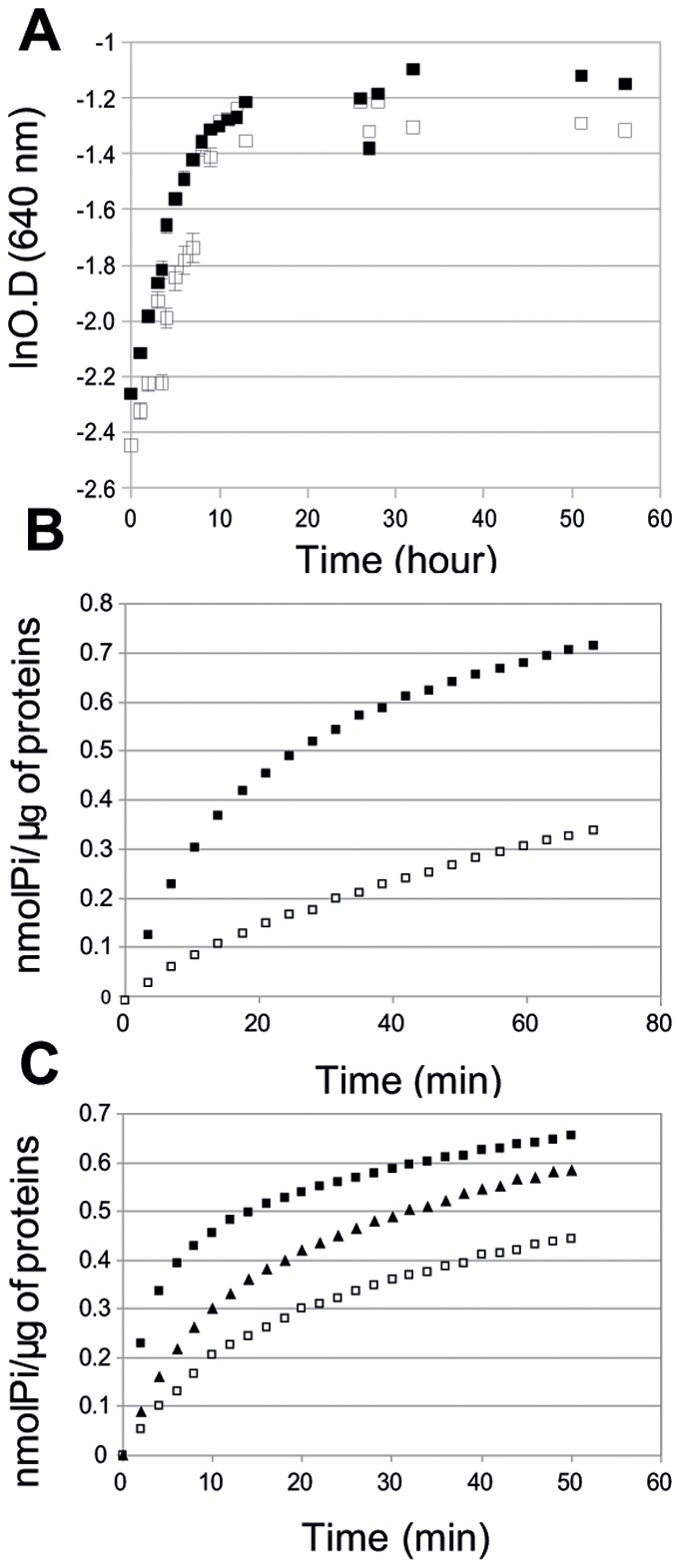
Growth and ATPase activity of *Mmm* T1/44 and the Δ619 mutant. **A**. Growth of *Mmm* T1/44 (▪) and Δ619 (□) in Hayflick medium at 37°C. **B**. Rate of release of Pi from ATP in the presence of membrane preparations from T1/44 (▪) and Δ619 (□). The figure shows representative results of five independent experiments. **C**. Rate of release of Pi from ATP in the presence of membrane preparations from T1/44 (▪) and Δ619 (□) transformed with the control plasmid pMYSO1, and Δ619 complemented with the MSC_0619 (α-like) and MSC_0618 (β-like) proteins, generated from the plasmid pCC1 (▴).

A previously obtained library of mutants generated by random insertion of the gentamycin resistance transposon Tn4001 in *Mmm* T1/44 [32] was screened for insertion in genes belonging to the Type 3 cluster. A mutant carrying Tn4001 inserted at position 307 of the *MSC_0619* gene (**Figure 7A**) was identified by PCR as described in the Materials and Methods. The insertion of the 3.7 kbp transposon leaded to a predicted truncated protein of 108 amino acids whereas the complete protein includes 505 amino acids. Comparative growth studies were performed for preliminary characterization of the mutant phenotype. The T1/44 and Δ619 strains were grown in pyruvate-supplemented Hayflick medium at 37°C and the optical density at 640 nm was recorded over a period of 60 h. The time courses for growth were very similar, except that the cell density observed in stationary growth phase was repeatedly slightly lower for the mutant strain (**Figure 8A**). Both strains were able to acidify the culture medium, the pH decreasing from an initial value of 7.4 to 5.4 for T1/44, and to 5.8 for the mutant strain at t = 30 h.

We assessed expression of the *MSC_0618-MSC_0626* genes in the Δ619 insertion mutant. As transcriptional analyses of the T1/44 provided strong evidence that the cluster containing genes *MSC_0618* to *MSC_0626* functioned as an operon, we expected the insertion of Tn4001 in *MSC_0619* to affect expression only of *MSC_0619* and *MSC_0618*, which is located immediately downstream from the gene carrying the inserted transposon. On a western-blot of membrane protein extracts from Δ619 cells, polyclonal antibodies directed against Protein 5 (MSC_0620) detected a band corresponding to a protein of the expected size for MSC_0620, indicating that this protein was produced in Δ619 (**Figure 7B**). However, MSC_0619 and MSC_0618 were not detected with the polyclonal antibodies. For confirmation of these results, we carried out LC-MS/MS analyses after separating the membrane-associated proteins by SDS-PAGE. This approach revealed the presence of the MSC_0620 to MSC_0626 proteins in the insertion mutant, but the absence of MSC_0619 or MSC_0618 (**Figure 7C**), consistent with the lack of expression of the two last genes of the cluster. This confirmed that the cluster *MSC_0618-MSC_0626* functions as an operon.

*In silico* analyses strongly suggested a F_1_-likeX_0_ structure for the proteins encoded by the Type 3 cluster. We therefore investigated the roles of the proteins of the cluster in *Mmm* further, by evaluating the total ATPase activity of membrane extracts from axenic cultures of T1/44 and Δ619. The isolation of membrane-enriched fractions by sonication may lead to an artificial general loss of membrane protein activity. We assessed the ability of the membrane-enriched fractions to generate hydrogen peroxide in response to glycerol addition, as a means of evaluating the metabolic activity of these fractions. Indeed, in *Mmm* after import and phosphorylation of glycerol, the synthesis of H_2_O_2_ requires the activity of the membrane located enzyme L-α-glycerophosphate oxidase GlpO. In this control experiment, the membrane-enriched fractions obtained from the T1/44 and Δ619 strains produced H_2_O_2_ with similar kinetics, with specific rates of H_2_O_2_ production varying from 5 to 6 µmol. mg protein^−1^. h^−1^ for both strains. This indicated that the fractions prepared from both strains had similar overall levels of metabolic activity. ATPase activities as determined by an *in vitro* colorimetric assay are shown **Figure 8B**. In this experiment, we assessed the generation, by washed membrane vesicles, of Pi following the addition of ATP. The membrane-enriched fractions of Δ619 displayed significantly lower (p<0.05) levels of total ATPase activity than those of T1/44. Activity in Δ619 was only 20% that of T1/44, indicating strong correlation between *MSC_0619* inactivation and the large loss of ATPase activity in membrane-enriched fractions. These data suggest that MSC_0618 and/or MSC_0619 proteins are involved in membrane-associated total ATPase activity. We tested this hypothesis, by carrying out a complementation assay. *Mmm* Δ619 was complemented with the pCC2 plasmid carrying *MSC_0618* and *MSC_0619*. Both α-like and β-like proteins were produced in the complemented strain as shown by LC-MS/MS (not shown). Complementation partially restored the total ATPase activity of membrane-enriched fractions (**Figure 8C**), thus confirming the involvement of MSC_0618 and MSC_0619 in the maintenance of total ATPase activity in isolated membrane fractions.

## Discussion

### Evolution of Type 2 and Type 3 clusters

In addition to the typical operon encoding Type 1 F_1_F_0_ ATPase, an analysis of mycoplasma genomes detected clusters of seven genes, four of which were predicted to encode proteins related to the α-, β-, γ- and ε-subunits of the F_1_F_0_ ATPase.

The phylogenetic reconstructions inferred from the α- and β-subunits and comparisons of genomic contexts showed a distribution of these clusters into two well supported groups, identified here as Type 2 and Type 3. F_1_-like sectors potentially assembled from these clusters shared a common ancestry with genuine F_1_F_0_ ATPase, N-ATPases and, more distantly, with other ATPases such as V-type ATPase and type 3 secretion systems. The relative positions of the branches corresponding to Type 1 (F_1_F_0_ ATPases), Type 1′ (N-ATPases), Type 2 and Type 3 ATPases remain unclear, but the monophyletic distributions of the Type 2 and Type 3 ATPases indicated that they had not recently emerged by sporadic duplications in particular mycoplasma species.

For Type 3 ATPases, clustering was not entirely consistent with the three well established phylogenetic groups of mycoplasmas (Spiroplasma, Pneumoniae and Hominis). Moreover, Type 3 clusters in the same genome were not always clustered on sister branches, and therefore cannot be assumed to have arisen through recent duplication events. Sequence variability and the evolutionary distances calculated for β-subunits suggested that Type 2 and Type 3 ATPases evolved more rapidly than Type 1 F_1_F_0_ ATPases. This more rapid evolution of Types 2 and 3 ATPases was particularly marked for Proteins 1, 2, 3, 4 and 5, as blast queries did not even detect reciprocal relatedness. All of these data suggested a complex evolutionary scenario including HGT and a remarkably fast evolution of protein sequences.

The distribution of Type 3 ATPases among mycoplasmas suggested that the ancestor of the Hominis branch had this specific ATPase. Nearly all other Type 3 ATPases found in mycoplasmas from other phylogenetic groups were highly related with homologs from species of the Hominis group. This suggested a scenario with spreading from this group to the others by HGT. Only one single Type 3 ATPase of the bird pathogen *M. gallisepticum* failed to show any closely related homolog in the Hominis group. However, even in this particular case, the phylogenetic tree indicated a close relationship with Type 3 ATPases from the Hominis group, consistent with a hypothetical acquisition from this group. Interestingly, the other Type 3 cluster in *M. gallisepticum* genome is highly degraded but very closely related to a complete cluster in *M. synoviae*, another bird pathogen. This suggests that a large deletion occurred during or after gene transfer into *M. gallisepticum*. The phylogenetic reconstructions and analysis of genomic contexts also suggested HGT of Type 3 ATPases between ruminant mycoplasmas (*M. agalactiae* and species from the mycoides group) and between human mycoplasmas (*M. hominis* and *U. parvum*). In these cases, gene exchanges may have been extended to a pair of genes, located upstream of the Type 3 cluster and directly repeated up to four times (*M. capricolum* subsp. *capricolum* and *M. mycoides* subsp. *capri*) at this locus. These genes encoded a transmembrane protein with a predicted bacteriocin motif and a lipoprotein with a serine-protease domain. Such genes might be involved in pathogenicity, but they were not found systematically in the vicinity of Type 3 clusters. Thus, despite the fact that they may have been exchanged during the same HGT event, the functional relationship between these two groups of genes remains fairly hypothetical.

Several genomes are now available for some species harbouring Type 3 clusters (*M. gallisepticum*, *M. hyopneumoniae*, *U. urealyticum/parvum*, *M. agalactiae*, *Mmm* and *M. mycoides* subsp. *capri*). In all cases, the Type 3 clusters were found at the same locus in all strains. This indicated that HGT involving Type 3 clusters pre-dated intra-specific diversification. This observation also suggested that Type 3 ATPase spreading by HGT is probably much less frequent than movements of typical mobile elements (IS, ICE, RM) which occurrences and localizations often vary from one strain to another. No clear co-localization of the Type 3 clusters and mobile elements could be shown. However, we noticed that modifications of the chromosomal structure frequently occurred near the Type 3 locus for mycoplasmas from the mycoides group, including insertion of an ICE (*M. mycoides* subsp. *capri* GM12) or an IS (*Mmm* PG1) and a chromosomal inversion (*M. capricolum* subsp. *capricolum*). In *M. agalactiae* strain 5632, an ICE was also located upstream of the Type 3 cluster. These observations suggested that Type 3 clusters may tend to localize within highly dynamic chromosomal regions.

The N-type ATPases were found in the archaea *Methanosarcina* spp., in various marine bacteria and in pathogenic *Burkholderia* spp [11]. By contrast to Type 2 and Type 3 ATPases, N-type ATPases were encoded by operons containing genes typical of both F_1_ and F_0_ subunits. The phylogenetic trees (**Figure 2**) confirmed their specific evolution from F-type ancestor. The N-type ATPase operons were always found in genomes in addition to the genuine F_1_F_0_ ATPase operon and an evolution including HGT was suspected. The presence of N-type ATPase operons on plasmids of marine bacteria is in accordance with this hypothesis. Thus, despite Type 2 and Type 3 ATPases are only distantly related to N-type ATPases, they share some features that might be significant of their biological role in the adaptation to the environment.

### Functionality and expression of Type 2 and Type 3 clusters

In most species, the genes encoding Type 3 ATPases were found to be intact, suggesting biological significance. However, mutants with transposons integrated into the genes of Type 3 clusters were obtained by random mutagenesis in *M. pulmonis* [8] and *M. arthritidis* [10]. We also isolated an *Mmm* mutant lacking α-like and β-like subunits. These data demonstrate that Type 3 ATPases are not essential for life in axenic media. Moreover, the *Mmm* Δ619 did not grow significantly slower than T1/44, in axenic medium. This suggests that Type 3 ATPases are not involved in the central metabolism of mycoplasmas, instead possibly playing an adaptive role during interactions with the host.

Proteomic data for *Mmm* indicated that the seven genes of the Type 3 cluster were expressed *in vitro*. In all other mycoplasma species for which transcriptomic or proteomic data are available, the Type 2 and Type 3 clusters were found to be expressed. This was the case in *M. mobile* (Type 2 and Type 3) [43], [48], *M. agalactiae* strain 5632 [49] and *Mycoplasma hyopneumoniae* [50], [51].

In *Mmm*, RT-PCR and bioinformatics analyses indicated that the genes of the Type 3 cluster were organized into an operon that also included several upstream genes. The predicted horizontal transfer of these seven genes as a single block is also consistent with the idea they are functionally associated. Transcriptomic data obtained by Oneal *et al.* for *M. hyopneumoniae* showed that the expression of six of seven genes was increased by norepinephrine [52]. This stress hormone is produced by mammalian hosts during bacterial infections. Several bacterial species have been shown to respond to norepinephrine by an increase in growth rate and induction of virulence factors [53], [54]. This work showed not only that these genes may be coregulated, but also that two genes of the Type 3 cluster displayed more than 2-fold activation. The biological significance of this activation remains unclear, but these results suggest that Type 3 ATPase gene clusters maybe regulated during host-pathogen interactions.

### The relationship between Type 2 and Type 3 ATPases and possible biological functions

The organizations of the cluster and the structural features of the predicted proteins strongly suggested a common ancestry for Type 2 and Type 3 predicted ATPases. Nevertheless, with the exception of highly conserved subunits, namely the α-like and β-like subunits, the predicted Type 2 and Type 3 cluster proteins displayed only very low level of sequence similarity. Moreover, specific 200–300 amino acid-long N-terminal extensions were observed on the β-like subunits of Type 2. These differences raised questions about the possible biological functions common to these ATPase types. Interestingly, six of the seven proteins of the *M. mobile* Type 2 ATPase were associated with an intracellular “jellyfish” structure [48] connected to the complex gliding machinery on the external side of *M. mobile* cells [55]. Type 2 α-like and β-like subunits were found to be located within the cell. Our data also suggested that Type 3 ATPases were present within the cells.

The Type 2 and Type 3 clusters each encode four proteins structurally related to subunits α, β, γ and ε that could assemble into a structure similar to the F_1_ sectors of F-type ATPases. This prediction is consistent with functional assays showing that the Type 3 cluster is associated with a membrane ATPase activity in *Mmm*. Thus, there seems to be a clear relationship between F_1_-like structures and ATP hydrolysis.

Two general functions have been associated with structures including proteins related to the α- and β-subunits of ATPases: (1) H^+^/Na^+^ pumps associated with ATPase activities (F-ATPases, N-ATPases and V-ATPases) and, (2) translocation systems (T3SS, T4SS and flagellum). *In silico* analysis of the proteins encoded by Type 2 and Type 3 clusters but not predicted to have an F_1_-like structure (i.e. Proteins 1, 2 and 5) identified no sequence or structural similarity to any protein involved in ion transport or translocation systems. Therefore, the biological significance of Type 2 and Type 3 ATPases remains largely unknown.

One first hypothesis regarding to the cellular function of the Type 2 and Type 3 ATPases is an ion transport activity, as documented for F_1_F_0_ ATPases and N-ATPases. However, this would imply that F_1_-like structures of Type 2 and Type 3 ATPases are associated with a membrane complex functionally equivalent to the F_0_ sector. The three remaining proteins encoded by both Type 2 and Type 3 clusters did not show any sequence or structural similarity with the subunits a, b and c that form the F_0_ sector of F-type ATPases. Protein 5 is a large, highly structured protein with three predicted transmembrane segments, suggesting it is anchored in the membrane with a main part of the protein being intracellular and potentially available for other interactions. Thus, Protein 5 could be the main part of a peripheral stalk analogous to that formed by a and b proteins of F_0_ sectors. In F_1_F_0_ ATPases, the peripheral stalk maintains the (αβ)_3_ complex, leading to the rotation of γ and the membrane-located c ring [56]. In V-Type ATPases, an analogous structure was found, despite any homology with F-type proteins was demonstrated. The existence of analogous structures in prokaryotes suggests that several structures have evolved to form a functional peripheral stalk. Whether this is the case for Protein 5 or not remains unclear. In many bacterial F_1_F_0_ ATPases, the juxtaposition of 12 proteins c form a ring inserted in the membrane, each c monomer containing two transmembrane segments. This ring is connected with subunits γ and ε. Twelve transmembrane helices were predicted in the Protein 1 of Type 2 and Type 3 ATPases. An evolutionary scenario involving duplications and fusions of gene encoding c protein is also tempting but we found no repeated motifs that could argue in favour of this hypothesis. The Protein 2 has the size of a delta subunit but, we found no sequence or structural similarity between those two. Therefore, the appealing hypothesis that Proteins 1, 2 and 5 could form a structure functionally analogous to F_0_ remains purely speculative.

A second hypothesis proposes that Type 2 and/or Type 3 F_1_-like structures would hydrolyse ATP to provide the energy required for a translocation system. This would bring these mycoplasmal ATPases closer to bacterial T3SS, T4SS and flagellum apparatus. The Type 2 and Type 3 ATPases are phylogenetically close to F_1_F_0_ ATPases, compared with these prokaryotic translocation systems. However, the possibility of an evolutionary convergence cannot be formally rejected. Interestingly, a translocation function was proposed by Nakane *et al* for the Type 2 α-like and β-like subunits of *M. mobile* [48]. In this scheme, Type 2 ATPase could be in charge of translocating gliding proteins. In favour of such proposal is the structure of Protein 1 which 12 transmembrane segments are reminiscent of transporter permeases.

In some evolutionary scenario, it has been proposed that ancestors of F- and V-type ATPases could have a translocation function, first of RNA then of proteins [57]. In such scenario, the γ subunit found in F_1_F_0_ ATPases would originate from a protein which translocation would have finally been blocked. Therefore, it might seem difficult to propose a translocation function for Type 2 and/or Type 3 ATPase, given that a γ-like protein was clearly identified. Nevertheless, a recent study of the FliJ protein that is essential for export of flagellum proteins in *Salmonella enterica* serovar Typhimurium showed a remarkable similarity with part of γ subunits of F-type ATPases [58]. Thus, a translocation function of molecules with high molecular weight (i.e. proteins, ADN or ARN) might not be ruled out for Type 2 and/or Type 3 ATPases.

Both *in silico* and experimental approaches indicated that Proteins 1 and 5 were anchored to the cytoplasmic membrane, suggesting a hypothetical X_0_ structure that might be physically connected to the F_1_-like structure. Furthermore, a possible interaction of F_1_-like structures with the F_0_ sector of genuine F_1_F_0_ ATPases cannot be ruled out. Indeed, in experimental conditions, the disruption of *MSC_0619* led to the loss of about 80% of the total ATPase activities of membrane fractions from *Mmm*. This may not accurately reflect the contribution of Type 3 ATPase activity *in vivo*, but it does suggest that Type 3 ATPases are able to modify significantly the global ATP consumption associated with mycoplasmal membranes. A role of α–like and β–like subunits in the structural organization of the F_1_F_0_ ATPase complex might account for the much lower total ATPase activity recorded for membranes isolated from the mutant lacking the F_1_-like components.

### Conclusion

In summary, we have shown that clusters of seven genes including four related to the F_1_ sectors of F_1_F_0_ ATPases are present in the genomes of many mycoplasma species, in addition to the typical F_1_F_0_ ATPase operon. Two types of potential ATPases were described, Type 2 and Type 3, both evolving rapidly, with a complex pattern of evolution for Type 3, involving probable HGT. Our studies confirmed that the Type 3 cluster present in *Mmm* encoded a functional membrane ATPase. This evolution of F_1_-like structures is specific of mycoplasmas, with no apparent equivalent in other bacteria. The biological functions of Type 2 and Type 3 ATPases remain unclear, but the remarkably rapid evolution of these F_1_-like structures and their probable association with membrane proteins unrelated to all known ion pump and translocation systems provides insights into the ability of mycoplasmas to recycle and modify universally conserved proteins for the development of novel functional associations.

## Supporting Information

Table S1 **Primers used in this study.**
(DOC)

Table S2 **Genes included in the Type 2 and Type 3 clusters.**
(DOC)

Figure S1 **Type 3 cluster region exchanged between bird mycoplasmas.** The complete Type 3 cluster in *M. synoviae* and the deleted form present in *M. gallisepticum* are indicated under the bracket. The xenologous regions are connected by yellow bands. The schematic diagram was composed from screenshots obtained from the MBGD database. HP, Hypothetical Protein.
(TIF)

Figure S2 **Conserved positions in Type 2 and Type 3 proteins.** Concatenated amino acid sequences of the clusters were aligned by MAFFT. Overview of the conserved positions over the cluster was obtained by Jalview. Genes from the ATPase cluster were represented as coloured boxes on the top of the diagram. Conserved positions were coloured as blue bars. Identity threshold for colouring was 50%. Red bars indicate genes boundaries.
(TIF)

Figure S3 **Secondary structure of Proteins 1 to 5 from** ***Mmm*** **.** (**A**) Protein 1 (MSC_0624) contained twelve transmembrane helices. (**B**) Protein 2 (MSC_0623) is a α-helix rich protein. (**C**) Protein 3 (MSC_0622) presented long N-terminus (amino acids 2 to 84) and C-terminus (amino acids 222 to 290) helices surrounding a mixed α-helix/β-strand region (amino acids 85 to 221). (**D**) Protein 4 (MSC_0621) contained eight strands (amino acids 3 to 90) followed by two helices (amino acids 96 to 143). (**E**) Protein 5 (MSC_0620) displays N-terminus (amino acids 1 to 190) and C-terminus (amino acids 460 to 770) regions rich in helices and a central region (amino acids 190 to 460) with both helices and strands. It was predicted anchored into the membrane through at least two transmembrane helices near the C-terminus (amino acids 707 to 768). (**F**) Two topology models for MSC_0620 were predicted. TMHMM (left) suggested that Protein 5 was mainly (amino acids 31 to 709) surface-exposed while it was predicted to be mainly cytoplasmic using TMpred (right). The panels (B–E) were composed from PSIPRED drawings.
(TIF)
