# Supplementary material for: Specific Evolution of F1-Like ATPases in Mycoplasmas
Source: PLoS One. 2012 Jun 7;7(6):e38793. doi: 10.1371/journal.pone.0038793 (PMC3369863; doi:10.1371/journal.pone.0038793)
Supplement: Table S1 — Primers used in this study. (DOC) [file pone.0038793.s001.doc]

Table S1. Primers used in this study

| **Name** | **Sequence (5’-3’)** | **Use^a^** | **Size (bp)^c^** |  |  |
| --- | --- | --- | --- | --- | --- |
| MSC620.3 | AGCAACAAGTGAGTATCAATCACTTAG | ID^a^ | 1190 |  |
| MT85.1 | ACAGTAATTGCGGGTGGATC | ID |  |
| MSC627.1 | GGTGGTGGAAGTGATCAGGC | RT PCR A (Fw)^b^ | 600 |  |
| MSC626.2 | GCAGCTAGTTCAGCATCAG | RT PCR B (Rev) |  |
| MSC626.1 | CTGGAGAAGGAAATCTTGAA | RT PCR C (Fw) | 783 |  |
| MSC625.1 | TCATTAAAACCGCTAAGACC | RT PCR D( Rev) |  |
| MSC625.2 | CTAATGGATGAGCCAAAACT | RT PCR E (Fw) | 711 |  |
| MSC624.3 | CTAAATCCATTAGTATATCAAAACA | RT PCR F (Rev) |  |
| MSC624.2 | AAATACCATCAACTCATGAAGTATTTG | RT PCR G (Fw) | 960 |  |
| MSC623.2 | TTGGAAAAATTTCAACACAAA | RT PCR H (Rev) |  |
| MSC623.1 | CACAAAAACAAACAAACTAGCGA | RT PCR I (Fw) | 763 |  |
| MSC622.2 | AATTCAAAGTTCTTTTGGTTGAAA | RT PCR J (Rev) |  |
| MSC622.3 | CGTGAAAAAGAAATTGAAGAAATT | RT PCR K (Fw) | 734 |  |
| MSC620.4 | CTTGTACCTGAACCACCATT | RT PCR L (Rev) |  |
| MSC620.5 | TTCAACAAACTTATCAAAAAGC | RT PCR M (Fw) | 831 |  |
| MSC619.9 | TTTTTGACCAATAGCAACAT | RT PCR N (Rev) |  |
| MSC619.6 | TCAGTTGATGGTAAGAAACAATTTG | RT PCR O (Fw) | 860 |  |
| MSC618.4 | TGGAACAATTGACATACGTG | RT PCR P (Rev) |  |
| MSC618.8 | CATATATTGGATCAGCTTTAG | RT PCR Q (Fw) | 600 |  |
| MSC617.1 | CAAGTTTAACTGAAGTTGC | RT PCR R (Rev) |  |
| MSC679.1 | CAGCTCAAGGAATCTTTTGT | RT PCR (Rev) | 788 |  |
| MSC679.2 | TTGTTTTCCATCAACATTCA | RT PCR (Fw) |  |
| PS1 | ATATATCCTCTAGAGTCGACCTGCAG | CL^c^ | 311 |  |
| PS2 | ATTATATCTAGA**CTCGAG**CTGCAGTTCAGATCTTCAATTTCC | CL |  |
| MSC618.5 | TAATA**CTCGAG**TTATAATCCTAATTCTTCATCGG | CL | 2883 |  |
| MSC619.10 | ATATA**CTCGAG**ATGAATATAAAAACAAATAGC | CL |  |

^a^ use of the primers: ID, screening of transposon-generated mutant library ; RT PCR, reverse transcriptase PCR assay ; CL, cloning of *MSC_0618-MSC_0619* genes (*Xba*I (underline) and *Xho*I (bold type).

^b^For RT PCR, letters A to R refer to the RT-PCR experiments (see Figure 7A) ; Fw, forward primer ; Rev, reverse primer.

^c^size (bp), expected size of the PCR product
