# Supplementary material for: Specific Evolution of F1-Like ATPases in Mycoplasmas
Source: PLoS One. 2012 Jun 7;7(6):e38793. doi: 10.1371/journal.pone.0038793 (PMC3369863; doi:10.1371/journal.pone.0038793)
Supplement: Table S2 — Genes included in the Type 2 and Type 3 clusters. (DOC) [file pone.0038793.s002.doc]

Table S2. Genes included in Type 2 and Type 3 clusters

| species | 1 | 2 | 3 (*atpG-like*) | 4 (*atpC-like*) | 5 | 6 (*atpA-like*) | 7 (*atpD-like*) |
| --- | --- | --- | --- | --- | --- | --- | --- |
| Type 2 cluster |  |  |  |  |  |  |  |
| *M. mobile* | MMOB1610 | MMOB1620 | MMOB1630 | MMOB1640 | MMOB1650 | MMOB1660 | MMOB1670 |
| *M. pulmonis* | MYPU_2290 | MYPU_2300 | MYPU_2310 | MYPU_2320 | MYPU_2330/MYPU_2340 | MYPU_2350 | MYPU_2360 |
| Type 3 cluster |  |  |  |  |  |  |  |
| *M. capricolum* subsp. *capricolum* | MCAP0353 | MCAP0354 | MCAP0355 | MCAP0356 | MCAP0357 | MCAP0358 | MCAP0359 |
| *M. mycoides* subsp. *mycoides* | MSC_0624 | MSC_0623 | MSC_0622 | MSC_0621 | MSC_0620 | MSC_0619 | MSC_0618 |
| *M. mycoides* subsp*. capri* | MMCAP2_0581 | MMCAP2_0580 | MMCAP2_0579 | MMCAP2_0578 | MMCAP2_0577 | MMCAP2_0576 | MMCAP2_0575 |
| M. agalactiae | MAG2880 | MAG2890 | MAG2900 | MAG2910 | MAG2920 | MAG2930 | MAG2940 |
| *M. hominis* | MHO_3180 | MHO_3170 | MHO_3160 | MHO_3150 | MHO_3140 | MHO_3130 | MHO_3120 |
| *M. arthritidis* | MARTH_orf250 | MARTH_orf251 | MARTH_orf252 | MARTH_orf254 | MARTH_orf255 | MARTH_orf258 ^a^ | MARTH_orf256 ^a^ |
|  | MARTH_orf505 | MARTH_orf503 | MARTH_orf502 | MARTH_orf501 | MARTH_orf500 | MARTH_orf499 | MARTH_orf498 |
| *M. mobile* | MMOB3020 | MMOB3010 | MMOB3000 | MMOB2990 | MMOB2980 | MMOB2970 | MMOB2960 |
| *M. conjunctivae* | MCJ_003410 | MCJ_003420 | MCJ_003430 | MCJ_003440 | MCJ_003450 | MCJ_003460 | MCJ_003470 |
| *M. hyopneumoniae* | mhp482 | mhp481 | mhp480 | mhp479 | mhp478 | mhp477 | mhp476 |
| *M. pulmonis* | MYPU_4440 | MYPU_4450 | MYPU_4460 | MYPU_4470 | MYPU_4480 | MYPU_4490 | MYPU_4500 |
|  | MYPU_5030 |  | MYPU_5020 | MYPU_5010 | MYPU_5000 |  |  |
|  |  | MYPU_7010 |  |  |  | MYPU_7000 | MYPU_6990 |
| *M. synoviae* | MS53_0155 | MS53_0156 | MS53_0157 | MS53_0157b *^b^* | MS53_0158 | MS53_0159 | MS53_0160 |
|  | MS53_0470 | MS53_0469 | MS53_0468 | MS53_0467 | MS53_0466 | MS53_0465 | MS53_0464 |
| *M. gallisepticum* | MGA_0477 | MGA_0480 | MGA_0482 | MGA_0484 | MGA_0485/ MGA_0487 *^c^* | MGA_0488 | MGA_0491 |
|  | MGA_0564^d^ |  |  |  |  |  | MGA_1321d ^d^ |
| *U. parvum* | UU048 | UU049 | UU050 | UU051 | UU052 | UU053 | UU054 |

^a^, MARTH_orf258 and MARTH_orf256 have changed orientation, see Figure 5B

*^b^*, this CDS was not annotated in the original work . It was annotated in the MolliGen database

*^c^*, from the genome sequence of *M. gallispeticum* , MGA_0485 and MGA_0487 are two parts of a pseudogene

*^d^*, truncated genes
