# Supplementary figures and images for: Specific Evolution of F1-Like ATPases in Mycoplasmas
Source: PLoS One. 2012 Jun 7;7(6):e38793. doi: 10.1371/journal.pone.0038793 (PMC3369863; doi:10.1371/journal.pone.0038793)

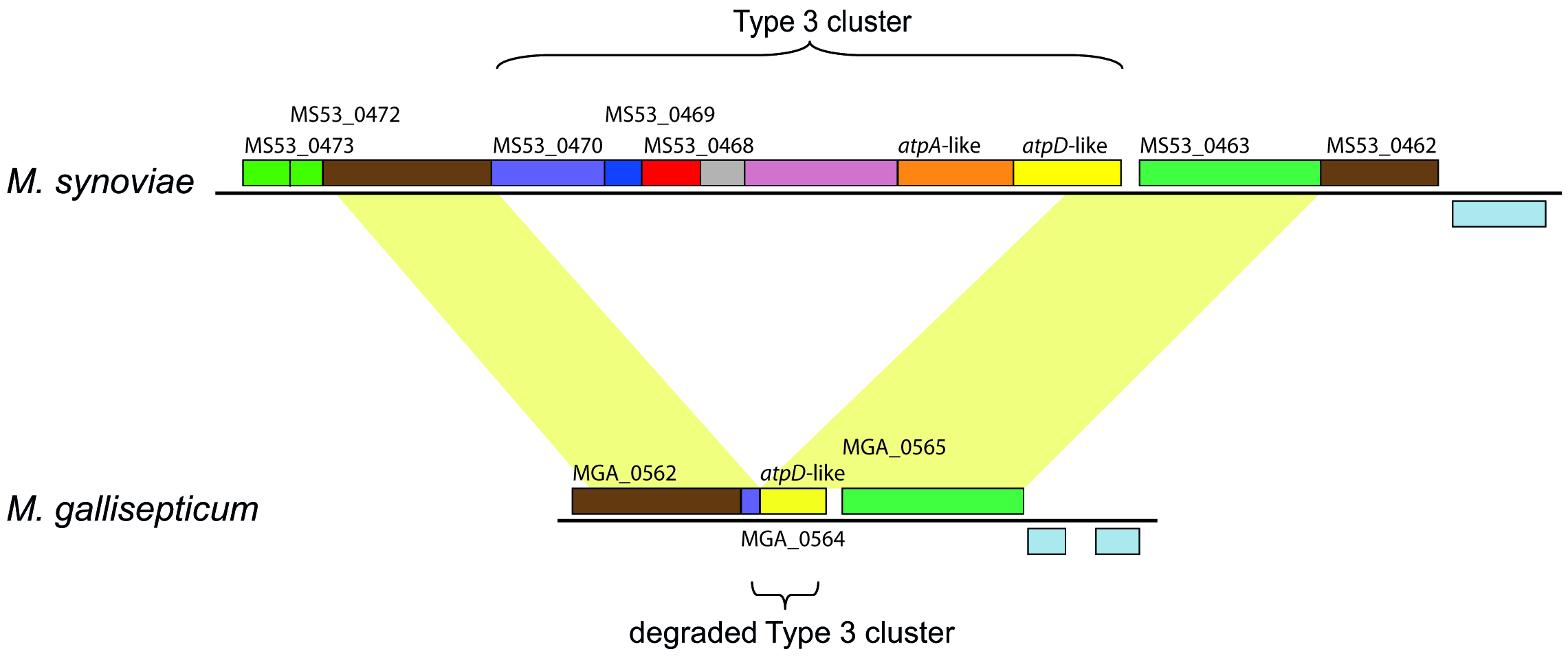

Supplement: Figure S1 — Type 3 cluster region exchanged between bird mycoplasmas. The complete Type 3 cluster in M. synoviae and the deleted form present in M. gallisepticum are indicated under the bracket. The xenologous regions are connected by yellow bands. The schematic diagram was composed from screenshots obtained from the MBGD database. HP, Hypothetical Protein. (TIF) [file pone.0038793.s003.tif]

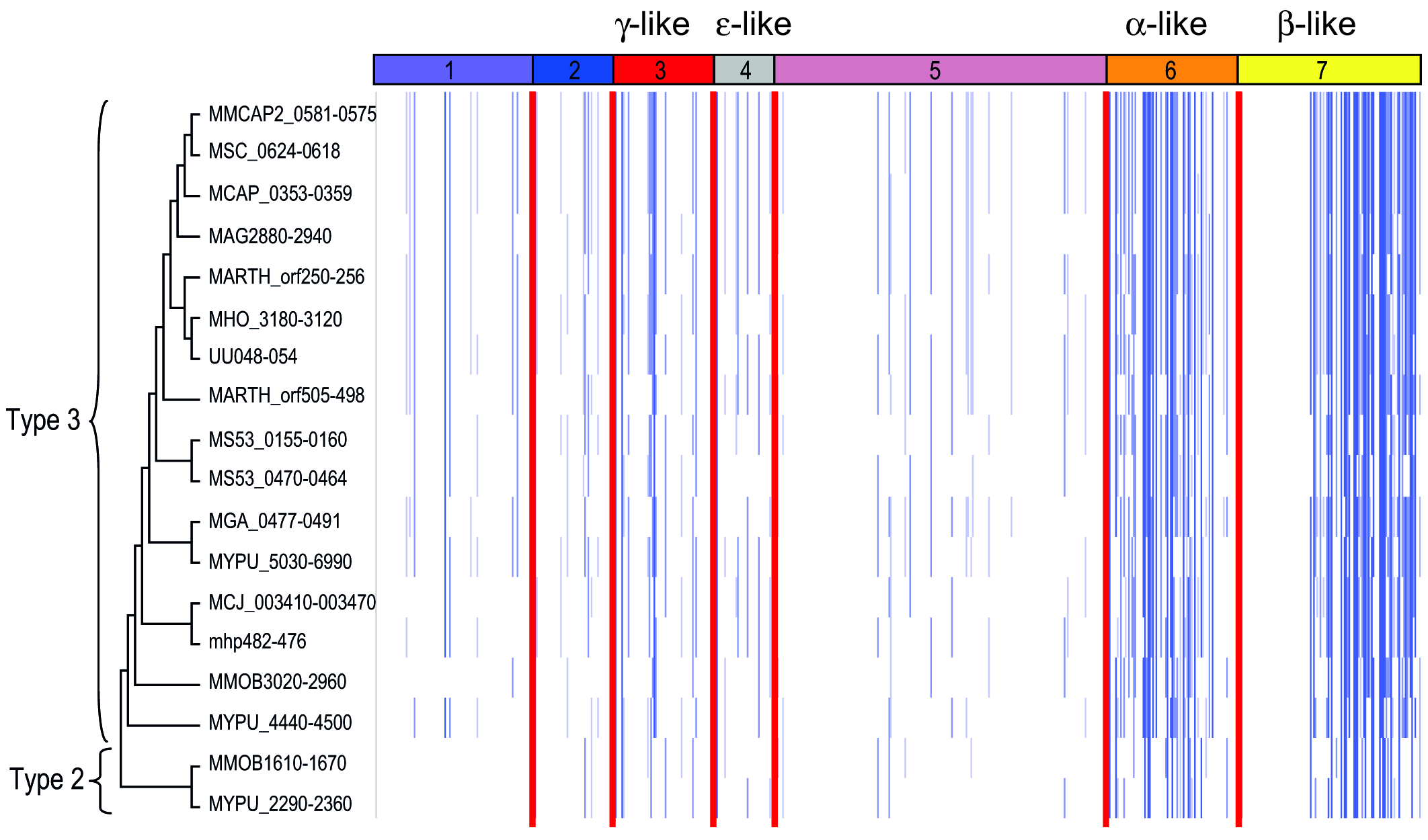

Supplement: Figure S2 — Conserved positions in Type 2 and Type 3 proteins. Concatenated amino acid sequences of the clusters were aligned by MAFFT. Overview of the conserved positions over the cluster was obtained by Jalview. Genes from the ATPase cluster were represented as coloured boxes on the top of the diagram. Conserved positions were coloured as blue bars. Identity threshold for colouring was 50%. Red bars indicate genes boundaries. (TIF) [file pone.0038793.s004.tif]

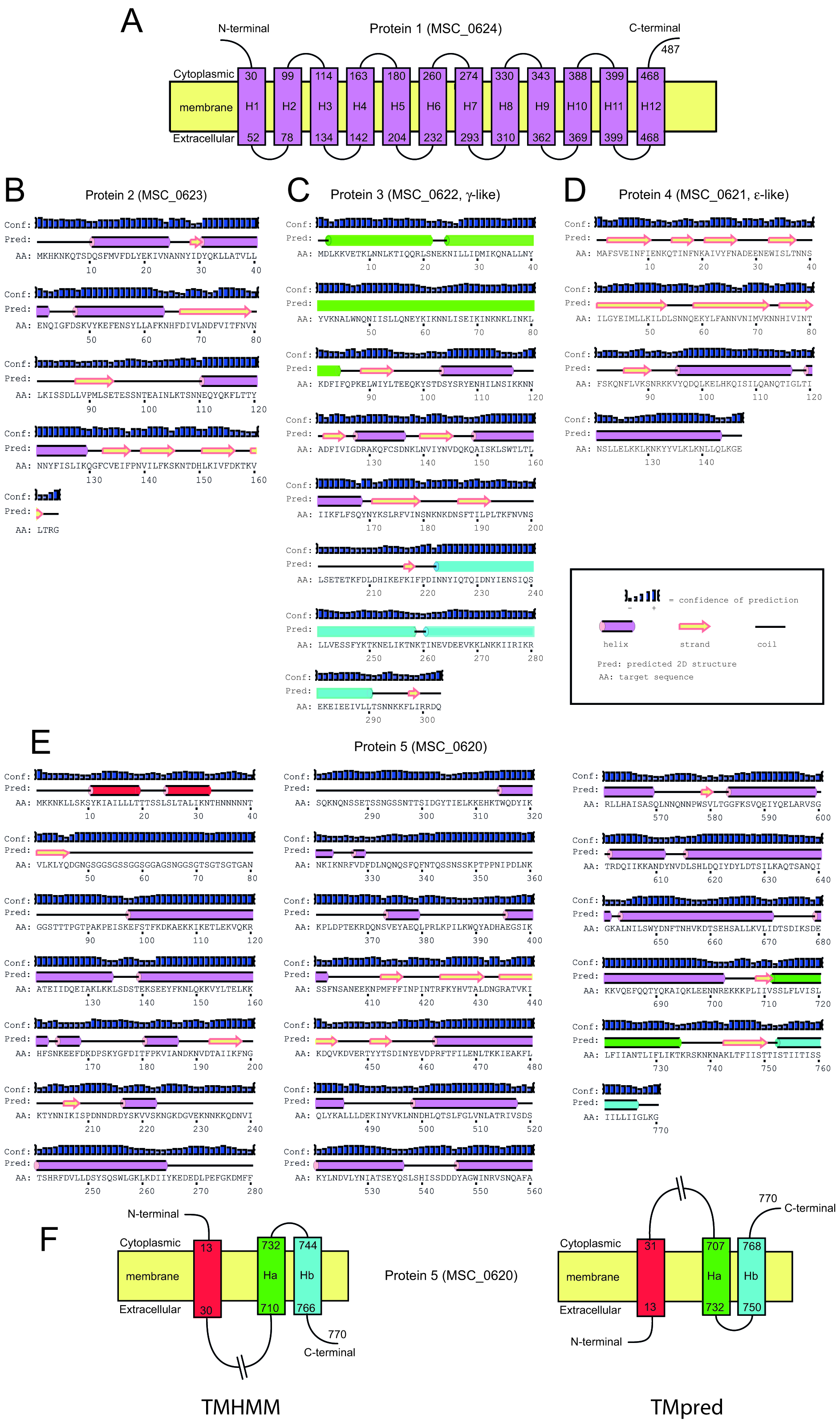

Supplement: Figure S3 — Secondary structure of Proteins 1 to 5 from Mmm . (A) Protein 1 (MSC_0624) contained twelve transmembrane helices. (B) Protein 2 (MSC_0623) is a α-helix rich protein. (C) Protein 3 (MSC_0622) presented long N-terminus (amino acids 2 to 84) and C-terminus (amino acids 222 to 290) helices surrounding a mixed α-helix/β-strand region (amino acids 85 to 221). (D) Protein 4 (MSC_0621) contained eight strands (amino acids 3 to 90) followed by two helices (amino acids 96 to 143). (E) Protein 5 (MSC_0620) displays N-terminus (amino acids 1 to 190) and C-terminus (amino acids 460 to 770) regions rich in helices and a central region (amino acids 190 to 460) with both helices and strands. It was predicted anchored into the membrane through at least two transmembrane helices near the C-terminus (amino acids 707 to 768). (F) Two topology models for MSC_0620 were predicted. TMHMM (left) suggested that Protein 5 was mainly (amino acids 31 to 709) surface-exposed while it was predicted to be mainly cytoplasmic using TMpred (right). The panels (B–E) were composed from PSIPRED drawings. (TIF) [file pone.0038793.s005.tif]
